# The biological pathways of Alzheimer disease: a review

**DOI:** 10.3934/Neuroscience.2021005

**Published:** 2020-12-16

**Authors:** Marco Calabrò, Carmela Rinaldi, Giuseppe Santoro, Concetta Crisafulli

**Affiliations:** Department of Biomedical and Dental Sciences and Morphofunctional Imaging, University of Messina, Italy

**Keywords:** Alzheimer disease, biological pathways, immune system, oxidative stress

## Abstract

Alzheimer disease is a progressive neurodegenerative disorder, mainly affecting older people, which severely impairs patients' quality of life. In the recent years, the number of affected individuals has seen a rapid increase. It is estimated that up to 107 million subjects will be affected by 2050 worldwide. Research in this area has revealed a lot about the biological and environmental underpinnings of Alzheimer, especially its correlation with β-Amyloid and Tau related mechanics; however, the precise molecular events and biological pathways behind the disease are yet to be discovered. In this review, we focus our attention on the biological mechanics that may lie behind Alzheimer development. In particular, we briefly describe the genetic elements and discuss about specific biological processes potentially associated with the disease.

## 1. Alzheimer's disease

Dementia is a broad category of neurodegenerative pathologies, whose main symptom is a decline in cognitive ability severe enough to interfere with activities of daily living. Among them, Alzheimer Disease (AD) is the most common type, accounting for 60% and up to 80% of the total Dementia cases.

According to the Global Burden of Disease Study, AD is one of the fastest rising diseases among the leading causes of death [1],[2]. About 10% of people of age 65 and older suffer from AD [3],[4]. Epidemiologic data in the last decade highlighted a sharp increase of AD incidence: According to the estimations, the number of AD subjects will increase from the 26.6 million worldwide in 2006 up to 107 million by 2050, with 16.5 in Europe [3],[4]. To note, 68% of the increase would be localized in the low- and middle-income countries [5].

AD has a progressive nature, and eventually leads to a severe cognitive decline. It mainly affects older people (>65 years old) even though earlier onset forms are also described in literature (10% of AD cases) [6]. From a clinical point of view, AD is staged in four different phases: preclinical, mild, moderate and late-stage (see Box 1). This classification is mainly based on cognitive decline.

Box 1. **AD Stages [6],[7]**

The clinical classification of AD is mainly based on the severity of cognitive decline and the histopathological alterations. Four stages are usually described:

**Preclinical:** This phases is often overlooked since no severe symptoms are present. It is usually classified as mild cognitive impairment. In this phase the earliest pathological changes begin, and hit entorhinal cortex (first) and hippocampus (later). From a symptomatologic point of view, subjects at this stage present mild memory loss with relative sparing of long-term memories. No significant impairment in their daily activities.

**Mild Alzheimer Disease:** In this phase cognitive symptoms start manifesting. The pathological alterations reach the cerebral cortex during this phase. From a symptomatologic point of view, along with memory loss, there is an inability to remember new information, forgetting things and appointments, followed by impairment in problem-solving, judgment and executive functioning. Also subjects manifest personality changes, mood swings and loss of spontaneity. Further, states of confusion and disorientation are commonly seen.

**Moderate Alzheimer Disease:** In this phase the symptoms severity increase further. The pathological damage further spreads to the areas responsible for language, reasoning and sensory processing (cerebral cortex). Other than an increased severity of symptoms from the previous phases, behavioral problems and social withdrawal tendencies begin to appear. This is followed by language disorder and impairment of visuo-spatial skills. Of note, subjects at this stage have trouble recognizing their own dears.

**Severe Alzheimer Disease:** In this phase, subjects completely lose their independency for daily activities. The pathological damage in this stage is believed to cover all of the cortex areas. From a symptomatologic point of view, the affected subjects cognitive abilities reach its lowest state, further systemic symptoms starts to appear, including difficulty performing learned motor tasks (dyspraxia), olfactory dysfunction, sleep disturbances, extrapyramidal motor signs like dystonia, akathisia, and parkinsonian symptoms.

To note, other staging systems were developed for AD, including the one introduced by Braak and Braak [7],[8]. This model is based on topographical staging of neurofibrillary tangles and divides AD progression into 6 stages. This Braak staging is an integral part of the National Institute on Aging and Reagan Institute neuropathological criteria for the diagnosis of AD [7].

The severity and the prevalence of AD promoted a flourishing research in order to develop strategies to confront this pathology.

In the 2002–2012 decade, over 240 drugs for AD were tested in publicly and privately funded clinical trials registered on the National Institutes of Health registry (clinicaltrials.gov) [9]. However, none of the pharmacologic treatments developed so far is able to cure or halt AD progression [10]–[16]. The two categories of approved drugs, Cholinesterase Inhibitors (tacrine, donepezil, rivastigmine, and galantamine) and partial N-methyl D-aspartate (NMDA) antagonists (memantine), only provide a temporary amelioration of symptoms [17]–[20]. For a brief summary, please see supplementary Box 1. Furthermore, these drugs have a variable efficacy among the subjects [21].

The absence of a definitive treatment is related to the lack of knowledge about the precise molecular mechanics and the events behind the disease [5].

Although the grade and type of symptoms may vary greatly from person to person [22], post mortem observations on AD subjects' Central Nervous System (CNS) evidenced some common features: (1) synaptic loss, (2) accumulation of abnormal neuritic plaques and (3) presence of neurofibrillary tangles [23]–[25]. The first lesions are believed to start accumulating 10–15 years before the onset of cognitive symptoms [26].

## 2. Physiopathological manifestations

Neuritic plaques are spheroid-like microscopic lesions characterized by a core of extracellular amyloid β peptide (Aβ) and surrounded by abnormal axonal endings. Aβ is derived from a large protein called amyloid precursor protein (APP). APP can be cleaved by the action of enzymes named α-, β-, and γ-secretase. In normal individuals, APP is firstly cleaved by α secretase and then γ secretase [27],[28]. The action of α secretase eliminates the risk of formation of an Aβ peptide.

In the neurons of the subjects suffering from AD, it is the β-secretase enzyme that acts to cleave the APP molecule instead of α secretase, and the resulting sAPPβ is released from the cell [29]. The sequential cleavage by β and then γ-secretase results in Aβ40, Aβ42 (β-amyloid 40 and β-amyloid 42) and C99 amino acid peptides [30]. The increased concentration of Aβ42 (from thereon Aβ) favors the formation of oligomers, which have neurotoxic properties [30]. These oligomers tend to cluster around meningeal and cerebral vessels and gray matter in AD. These deposits coalesce to form the miliary structures that are known as plaques [7]. Interestingly, recent data suggest the importance of cholesterol for the γ-secretase cleavage of amyloid precursor protein, the last step of Aβ formation [32].

Regarding neurofibrillary tangles, they are fibrillary intracytoplasmatic structures in neurons formed by a protein called Tau. Physiologically, the primary function of Tau protein is to stabilize axonal microtubules. Usually, Tau is bound to microtubules and presents a certain number of phosphate molecules attached to it. The phosphorylation mechanics of Tau are altered in AD for reasons not entirely understood. This alteration leads to an abnormal increase of the phosphorylation, which in turn causes the detachment of Tau molecules from microtubules. Detached hyper-phosphorylated Tau proteins tend to assemble to form filamentous structures known as paired helical filaments, which in turn aggregate in the insoluble neurofibrillary tangles [33],[34]. Post mortem analyses on AD subjects demonstrated that few of the many different types of neurons in the brain develop abnormal Tau aggregates [35],[36]. They are all projection neurons with high concentrations of neurofilaments whose axons are excessively long and thin compared to the size of their soma [36]. Analyses on AD subjects on various stages evidenced that neurofibrillary tangles start to form in the transentorhinal cortex, spread to the hippocampus, and then progress to cover the cerebral cortex at later phases [37]–[39].

Another typical feature of AD is the granulo-vacuolar degeneration observed in the hippocampal pyramidal cells. This degeneration is likely associated to the cognitive decline: alterations of cognition processes are correlated to the decrease in density of presynaptic buttons from pyramidal neurons in laminae III and IV [7]. The decrease of synaptic buttons is likely associated to the vascular degeneration. Indeed, the risk of dementia is increased fourfold with subcortical infarcts; also the presence of a cerebrovascular disease exacerbates the degree of dementia and its rate of progression. However, the mechanisms behind are not fully determined [7].

## 3. Genetics

Despite a large part of its biological background is not characterized, AD has a strong genetic correlation with 3 genes: APP and the genes for the presenilin 1 (PSEN1) and presenilin 2 (PSEN2) proteins. Alterations within these genes are directly correlated with plaques formation. Literature data demonstrated that subjects inheriting mutations within APP or PSEN1 genes are guaranteed to develop AD, while those inheriting mutations within PSEN2 gene have a 95 percent chance of developing the disease [40]. The cases of AD caused by alterations within the three genes are labeled as autosomal dominant familial AD, because of its inheritance model. It generally develops within 60 years of age, sometimes as early as 30 years of age [5]. For this reason, it is also often indicated as early onset AD (EOAD). This form presents a clear molecular background and it's easily recognized since it runs in families. Up to 5% of all AD cases are of this type [41].

The largest part of AD cases (more than 95% AD) presents a sporadic manifestation and usually manifests between the 60 and 65 years of age (Late onset AD or LOAD). This form has a more cryptic genetic background [41]:

Both EOAD and LOAD may occur in people with a positive family history of AD. About 60% of EOAD subjects have relatives affected by AD [42]. Around 13% of these subjects present an autosomal dominant inheritance with at least 3 generations affected [42]. EOAD cases may also occur in LOAD families [43].

Regarding the risk factors, contrary to EOAD single-gene inheritance, virtually all LOAD cases likely involve multiple susceptibility genes and environmental factors [2],[43],[44]. Although none of them can be labeled as causative, they are associated with an increased risk of developing the disease. Excluding the known three causative genes of EAOD form, only one other gene evidenced a strong association with AD risk: the Apolipoprotein E gene (APOe).

APOe is the gene encoding for the Apolipoprotein E, whose function is to bind lipids and sterols and transport them through the lymphatic and circulatory systems. In particular, APOe is in charge of cholesterol transport in the brain [45],[46]. Several studies associated the isoform e4 of this protein to an increased risk of developing AD [47]–[50]. At least one APOe4 allele is found in about 15–25% of the general population and can reach 50% in AD population [50],[51]. The fine molecular mechanisms behind the risk increase operated by APOe4 are not completely characterized, but seem to be related to the formation of neurofibrillary tangles [52],[53] and amyloid clearance processes [54],[55]. Also, this isoform alters multiple processes [56], in particular lipid metabolism [57],[58]. The e4 isoform is structurally unstable compared to other isoforms and thus it is not able to perform its functions normally. Further, the loss of cysteine residues decreases its antioxidant potential [59] and may lead to an increased production of oxidant molecules like malondialdehyde and hydroxynonenal (products of lipid peroxidation), as observed in APOe4 allele carriers [60],[61]. Data obtained from cell cultures evidenced how APOe4 promotes oxidative stress and the generation of neurotoxic fragments which impairs mitochondrial activity [62]–[64]. Furthermore, a reduction in cerebral glucose utilization was observed in brain regions typically affected by AD [65],[66].

Following the discovery of APOe4 association with LOAD, a number of studies investigated other genes involved in the same molecular cascades of APOE. More than 500 candidates were proposed in the last two decades (see Table 1 and [67],[68] and references therein). They showed correlations with Tau phosphorylation, vacuolar sorting proteins, metallo-proteins, glucose and insulin metabolism, nitrous oxide synthesis, oxidative stress, growth factors, neurodevelopment, pruning-, inflammation- and lipid-related pathways. The blooming of association studies on new genes led to the creation of a genetic database (www.alzgene.org) by Bertram and colleagues [69]. The site collects numerous publications regarding genetic data of AD and several genes appear to affect AD risk. Table 1 reports the top ranked genes involved in the risk of AD (from Alzgene.org). It has to be noted that these genes are believed to have a limited effect on the overall prevalence of AD because they are rare or only slightly affect (in total a ∼20% increase or decrease) AD risk [69].

**Table 1.** Genes significantly associated with Alzheimer's Disease (Retrieved from alzgene.org).

Gene Name	Extended Name	Brief summary (exert from genecards.org)	References
Genes associated with Early Onset Alzheimer's Disease			
APP	Amyloid β Precursor Protein	This gene encodes for a cell surface receptor and trans membrane precursor protein that is usually cleaved by α, β and γ secretases to form a number of peptides. The peptides are overall involved in functions relevant to neurite growth, neuronal adhesion and axonogenesis. In particular, some of them promote transcriptional activation, while others seem to be the basis for amyloid plaques formation. In addition, two of the peptides are antimicrobial peptides, having been shown to have bactericidal and antifungal activities. Mutations in this gene have been implicated in autosomal dominant Alzheimer.	[27],[70]–[73]
PSEN1	Presenilin 1	This gene encodes for the catalytic subunit of the γ-secretase complex, which is involved in the cleavage of integral membrane proteins, including APP. It plays a role in Notch and Wnt signaling cascades and regulation of downstream processes via its role in processing key regulatory proteins. It stimulates cell-cell adhesion and is required for normal embryonic brain and skeleton development. Alterations of this subunit were correlated with autosomal dominant Alzheimer.	[74]
PSEN2	Presenilin 2	This gene encodes for the catalytic subunit of the γ-secretase complex, which is involved in the cleavage of integral membrane proteins, including APP. Two alternatively spliced transcript variants encoding different isoforms of PSEN2 have been identified. It requires the other members of the γ-secretase complex to have a protease activity. It may play a role in intracellular signaling and gene expression or in linking chromatin to the nuclear membrane. It may function in the cytoplasmic partitioning of proteins.	[75]
Genes related to Late Onset Alzheimer's Disease			
ABCA7	ATP Binding Cassette Subfamily A Member 7	This gene encodes for a member of the ABC1 subfamily. Members of the ABC1 subfamily comprise the only major ABC subfamily found exclusively in multicellular eukaryotes. This transporter is often found in myelo-lymphatic tissues. The function of this protein is not entirely understood, but it is believed that it may have a role in lipid homeostasis in cells of the immune system, and in phagocytosis mechanics.	[76],[77]
ADAM10	ADAM Metallopeptidase Domain 10	This gene encodes an ADAM family member whose role is related to its cleaving properties. Interact with several proteins, including TNF-α and E-cadherin. It is also involved with constitutive and regulated α-secretase cleavage of APP. Finally, contributes to the normal cleavage of the cellular prion protein.	[78]
APOE	Apolipoprotein E	The protein encoded by this gene is a major apoprotein of the chylomicron and is essential for the normal catabolism of triglyceride-rich lipoprotein constituents. This protein Mediates the binding, internalization, and catabolism of lipoprotein particles and can serve as a ligand for the LDL (Apo B/E) receptor. Mutations in this gene result in an impaired clearance of chylomicron and VLDL remnants. Further, several evidences in literature associate specific APOE isoforms with an increased risk of Alzheimer disease.	[79],[80]
BACE1	β-Secretase 1	This gene encodes a member of the peptidase A1 family of aspartic proteases. This trans membrane protease catalyzes acts on APP. In particular, it leads to the generation and extracellular release of β-cleaved soluble APP, and a corresponding cell-associated C-terminal fragment which is later released by γ-secretase.	[81],[82]
BIN1	Bridging integrator 1	This gene encodes several isoforms of a nucleocytoplasmic adaptor protein which are expressed in several different tissues. Of note, isoforms expressed in the central nervous system may be involved in synaptic vesicle endocytosis. This gene has been identified as one of the most relevant risk locus for late onset Alzheimer's disease (LOAD).	[83]–[86]
C9ORF72	Chromosome 9 Open Reading Frame 72	The protein encoded by this gene plays an important role in the regulation of endosomes trafficking. It may regulate autophagy-related mechanics. Also, it regulates actin dynamics in motor neurons by inhibiting the GTP-binding activity of ARF6, leading to ARF6 inactivation. Positively regulates axon extension and axon growth cone size in spinal motor neurons. It plays a role within the hematopoietic system in restricting inflammation and the development of autoimmunity.	[87]
Genes related to Late Onset Alzheimer's Disease			
CD33	CD33 Molecule (Sialic Acid-Binding Ig-Like Lectin 3)	This gene encodes for a protein associated with Hematopoietic Stem Cell Differentiation Pathways and Lineage-specific Markers and Innate Immune System. In the immune response, it may act as an inhibitory receptor upon ligand induced tyrosine phosphorylation by recruiting cytoplasmic phosphatase(s) via their SH2 domain(s) that block signal transduction through dephosphorylation of signaling molecules.	[88]–[90]
CLU	Clusterin	The protein encoded by this gene is a secreted chaperone seemingly involved in several basic biological events including cell death, tumor progression, and neurodegenerative disorders. Its action inhibits formation of amyloid fibrils by APP, APOC2, B2M, CALCA, CSN3, SNCA and aggregation-prone LYZ variants.	[48],[91]–[93]
CR1	Complement Receptor 1	The gene encodes a monomeric single-pass type I membrane glycoprotein. The protein mediates cellular binding to particles and immune complexes that have activated complement.	[94],[95]
FUS	FUS RNA Binding Protein	This gene encodes a multifunctional protein component of the heterogeneous nuclear ribonucleoprotein (hnRNP) complex which is involved in pre-mRNA splicing and the export of fully processed mRNA to the cytoplasm. Its action influence gene expression, maintenance of genomic integrity and mRNA/microRNA processing.	[96]
GRN	Progranulin	This gene encodes for the 88 kDa precursor protein, progranulin. Cleavage of the signal peptide produces mature granulin which can be further cleaved into a variety of active, 6 kDa peptides. Granulin family members are important in normal development, wound healing, and tumorigenesis. Granulins have possible cytokine-like activity. They may play a role in inflammation, wound repair, and tissue remodeling.	[97]
LRRK2	Leucine-rich repeat kinase 2	The protein is present largely in the cytoplasm but also associates with the mitochondrial outer membrane. It is mainly involved in autophagy related mechanics. Of note, this protein seems to regulate neuronal process morphology in the central nervous system. Also, it plays a role in synaptic vesicle trafficking.	[98],[99]
PICALM	Phosphatidylinositol Binding Clathrin Assembly Protein α-synuclein	This gene encodes a clathrin assembly protein, which recruits clathrin and adaptor protein complex 2 (AP2) to cell membranes at sites of coated-pit formation and clathrin-vesicle assembly. Alterations within this gene are associated with Alzheimer.	[100]–[102]
SNCA	Synuclein Α	This gene may serve to integrate presynaptic signaling and membrane trafficking, in particular in the regulation of dopamine release and transport. It reduces neuronal responsiveness to various apoptotic stimuli. It has been found that SNCA peptides are a major component of amyloid plaques in the brains of patients with Alzheimer's disease. Also, it seems involved in the fibrillization of microtubule-associated protein Tau.	[103],[104]
Genes related to Late Onset Alzheimer's Disease			
SORL1	Sortilin Related Receptor 1	This gene encodes protein that belongs to at least two families: the vacuolar protein sorting 10 (VPS10) domain-containing receptor family, and the low density lipoprotein receptor (LDLR) family. Its role is likely related to endocytosis mechanics. SORL1 seems to be also associated with Alzheimer endophenotypes such as abstract thought, verbal memory, total brain volume, and white matter hyperintensities among people AD free. Regarding its mechanics SORL1 is believed to act through the regulation of the APP-containing endocytic vesicles trafficking.	[86],[105]–[108]
MAPT	Microtubule Associated Protein Tau	This gene encodes the microtubule-associated protein Tau (MAPT). MAPT transcripts are differentially expressed in the nervous system, depending on stage of neuronal maturation and neuron type. It promotes microtubule assembly and stability, and might be involved in the establishment and maintenance of neuronal polarity. MAPT gene mutations have been associated with several neurodegenerative disorders such as Alzheimer's disease.	[109]–[111]
TARDBP	TAR DNA Binding Protein	This protein is involved in the regulation of CFTR splicing. It seems to be related to microRNA biogenesis, apoptosis and cell division. Hyperphosphorylation of this protein has recently been described in association with cognitive impairment, especially in the context of Alzheimer's disease pathology.	[112],[113]
TREM2	Triggering Receptor Expressed On Myeloid Cells 2	This gene encodes a membrane protein that forms a receptor signaling complex with the TYRO protein tyrosine kinase binding protein. It is involved in immune response and in chronic inflammation. Defects in this gene are a cause of polycystic lipomembranous osteodysplasia with sclerosing leukoencephalopathy (PLOSL). Inactivating mutations of TREM2 have been associated with an autosomal recessive form of early-onset dementia in literature.	[114],[115]

Several other candidates and loci have been identified so far but replication analyses encountered some difficulties. Also, their contribute towards AD risk is deemed inferior to the genes of Table 1. Of them we want to report HLA-DRB5/DRB1, PTK2B, SLC24A4, RIN3, INPP5D, MEF2C, NME8, ZCWPW1, CELF1, FERMT2, CASS4, EPHA1, CD2AP, MS4A, CUGBP2, ATP5PD, MTHFD1L, GAB2, MEOX2 and PCDH11X [68],[77],[83],[89],[116]–[124].

Finally, Environmental factors like female gender [125], older age [126], low education level [127], smoking habit [128], obesity [129] and diabetes mellitus [129] increase the risk of AD. It is noteworthy to cite metals' levels as risk factors of AD. It was pointed out how metal homeostasis may be one of the processes which lead to the conformational changes of amyloidogenic proteins in AD [130]. Furthermore, while the variation of metals' concentrations is mainly environmental, alterations of protein transporters in the Blood Brain Barrier such as DMT1 or Transferrin may also alter metal homeostasis in the brain [131].

## 4. Molecular pathway contribution to AD risk

The number of genetic factors described are important contributors to AD, but even those cannot fully explain the totality of AD cases. Rather than single genes, a better approach would be investigating AD as an event related to alterations affecting entire biological pathways. A plethora of mechanisms, including neuroinflammation [132], oxidative stress [133],[134], defects in mitochondrial dynamics and function [135], cholesterol and fatty acid metabolism as well as glucose energetic pathways impairments in the brain [136],[137], autophagy failure [138] and other less studied mechanisms have been proposed to contribute to AD. In this section we will report a brief panoramic discussion on these mechanisms. It has to be noted that while the various processes are discussed separately, they are strictly linked with each other and often work in a synergic way in the CNS.

## 5. Chronic inflammation and immune system

Among the processes thought to be involved with AD, inflammation is one of the main biological mechanisms considered. Inflammation in the brain, is a well-established (acute) process which defend the body against infection, toxins and injury. When the delicate equilibrium between pro- and anti-inflammatory signaling is disrupted, it results in chronic (neuro)inflammation [139]–[141].

Multiple literature data pointed out that neuro-inflammation (and pruning, as discussed below) has a role in the neurodegeneration processes commonly observed in AD [142]–[146].

Although, neuroinflammation is not typically associated to AD onset on its own, it plays a key role in increasing the severity of the disease by exacerbating Aβ and Tau pathologies [147].

A prolonged neuroinflammation state increases the concentration levels of proinflammatory cytokines in the microenvironment. The increase of cytokines triggers several potentially harmful effects: it induces mitochondrial stress in neurons, either directly or indirectly, including via Aβ signaling. It also increases Oxidative stress [148]–[150] and Blood-Brain Barrier (BBB) permeability which likely influence AD progression [151],[152]. Furthermore, increased cytokines levels can influence other processes potentially related to AD. For instance, IL-18 increases the levels of Cdk5 and GSK-3β, which are involved in Tau hyperphosphorylation [153]. Also, the activation of Cdk5 pathway causes Golgi fragmentation, neuronal and mitochondrial fragmentation. Interestingly, the inhibition of Cdk5 was proven to improve AD subjects' conditions [154]–[156].

Several processes an natural compounds can trigger immune responses and deviate this process from its physiological behavior. In particular, there has been a lot of focus recently on the complement system.

The complement system facilitates the immune system' response. It's excessive activation exacerbates AD symptoms [157], since it influence Aβ, Tau, and APOE4 interaction in AD [158],[159] (for more details see [160]). Cholesterol also contributes to AD pathogenesis by inducing interleukin 1β production through cytoplasmic sensor NLRP3 [161]. Strictly related to the immune function, viral infections, in particular Herpes Viruses (HSV1, HSV2), are also associated with AD. In particular, this type of infection is believed to potentially be an import risk factor for AD onset. Indeed, HSV1 (oral herpes) and HSV2 (genital herpes) can trigger Aβ aggregation, and their DNA is common in Aβ plaques. HSV1 reactivation is associated with Tau hyperphosphorylation and possibly Tau propagation. Recurrent reactivation may produce neuronal damage and AD pathology. This data open for a potential viral hypothesis of AD, where an increased risk may follow neurotropic infection [162],[163]. The main players behind neuroinflammation and immune processes, are the non-neuronal cells that populate the brain (Neuroglia) [144],[164]. These once “supporting” cells were indeed demonstrated to have an important role in the regulation of Brain processes, including protection, neurotransmission and microenvironment homeostasis [165],[166]. Physiologically, these cells have the ability to phagocytize Aβ proteins, thus acting against AD-related Aβ accumulation [167]. However, this protective function can be inhibited by increased levels of inflammatory cytokines such as IL-1β, TNF-α and IFN-γ [140],[168]. Neuroglia is composed by different cell types. Box 2 briefly reports their potential role in association with AD.

Box 2. **Non-Neuronal Cells**

The continuous research on AD and brain in general, demonstrated how the physiological functions of SNC are not linked to neurons functions only. Rather, it was demonstrated that the once “supporting” cells types present in SNC have an important role in the regulation of Brain processes, including neurotransmission and microenvironment homeostasis [165],[166]. Alterations of their function may profoundly impact the physiological functions of the brain, and a large amount of literature data linked these alteration to several pathological states, including AD.

**Microglia:** Microglia are the resident phagocytes and innate immune cells of the brain, protecting from SNC from infections and also regulate the homeostasis. Over the past few decades, there has been an increased interest in microglia since the discovery of its involvement in synaptic plasticity dysfunctions. In particular, an alteration of the physiological functions of the cells has potentially harmful consequences for neuronal function, eventually leading to cognitive loss and behavioral deficits [169]. Also, synaptic plasticity has been demonstrated to be a key mechanics which is intimately linked with Aβ formation and with the long-term potentiation (LTP) pathways in AD [170],[171]. Recent data support a possible role of microglia in AD risk, and it is now widely accepted that clustered populations of reactive microglia are hallmarks of AD. Microglia cells in the brain are usually in an inactivated state. In this state they are still able to perform some control functions [172]–[174], but do not seem to influence AD-related processes. Their activation, though, can trigger several molecular processes that correlate with an increased risk of AD. In particular. when activated microglia may trigger pro-inflammatory (M1 state) or anti-inflammatory (M2 state) processes [175]. M2 state seems to play a role in neuroprotective-related processes. The action of microglial cells in M1 state instead, are linked to AD development since it promotes neuroinflammation through the secretion of pro-inflammatory cytokines. As described before, neuroinflammation is detrimental for neurons and may lead to neurodegenerative processes. Also, cells in M1 state may release ROS, thus increasing the OS in the micro-environment [176]. As such, M1 microglia cells may also act as a bridge of different processes both related with AD. Several studies have also focused on the existent relationship of microglia with Aβ plaques formation and Tau hyper-phosphorylation, evidencing an interesting correlation with the main events of AD development [169]. However, little is known about microglial activities in early stage AD and in particular, on its role in synaptic dysfunction [169].

**Astrocytes:** like microglia cells, Astrocytes are one of the subtypes composing neuroglia. Once believed to carry out only a support function to neurons, it is now known that Astrocytes can influence neuronal and vascular function. Accordingly, the functional consequences of astrocyte dysfunction in AD can be severe.

It was evidenced in literature that astrocyte action is important in the onset and development of neurodegenerative events, including the ones related to AD pathology [177]. It has to be noted, though, that they role is likely secondary to other alterations, since it seems that astrocytes have a neuroprotective function in the brain and are activated (reactive astrogliosis) in critical cases in order to limit neural damage in the brain [178]. However, chronic activation ultimately lead to imbalances “exacerbating cellular pathology and behavioral outcomes” [177]. In particular, literature data demonstrated that several events related to AD, like Aβ accumulation, synaptic dysfunction, neuronal energy metabolism and neuroinflammation are strictly linked to astrocyte action [179]. From this point of view, astrocyte may be considered as another bridge that link together the several processes related to AD development. Furthermore, it was evidenced that the role of astrocytes in controlling neurovascular interactions may be correlated with brain hypoperfusion and/or impaired Functional hyperemia, and both events are commonly observed in AD subjects [177]. This suggest an association between AD alterations and neurovascular functions. Consistently, it is interesting to note that Aβ accumulation seems to be mainly localized in brain areas with reduced cerebral blood flow [180]–[182]. Still, it has to be noted that the reduced vascularization may be a consequence of Aβ accumulation rather than a cause.

**Neutrophils:** Recently, some interesting literature data demonstrated neuroglial cells are not the only cell types that can be correlated with AD risk. Recent studies observed that the blood circulating immune cells, the neutrophils, seems to promote AD pathogenesis by triggering neuroinflammation-related AD events [183]. These cells are key regulators of the immune system because they communicate and interact with adaptive immune system cells during infections, chronic inflammation and autoimmune diseases [184],[185]. The mechanics behind the neutrophil-dependent damage in AD are not entirely understood, especially because they should not be able to pass BBB. According to this, their effect should be indirect, through the increase of pro-inflammatory cytokines, or subordinated to BBB damage. The recently discover of Neurotrophils extracellular traps (neutrophils mechanics of defense against infection) [183] in AD models, added a new potential way for the cells to influence AD-related mechanics (we recommend to consult [183] for further details).

## 6. Oxidative stress

Oxidative stress (OS) has been widely recognized as a prodromal factor associated to AD [186]. Cell control on OS is particularly important to maintain the balanced microenvironment needed for multiple biological processes, from bioenergetics to other essential functions such as vesicle transport or post-transcriptional modulations [187]. Neurons in particular are very sensible to OS as their normal antioxidant content is low and their membranes are rich of polyunsaturated fatty acids [188].

Multiple causes, mainly related to mitochondrial dysfunctions and energy metabolism impairments (as it will be described later in the text) can increase cellular OS. Even the natural process of aging is believed to physiologically increase OS with time [189]. At neuronal level, oxidative damage related to age [190],[191] strongly impairs the synaptic components involved in neuronal plasticity [189],[192], cytoskeletal dynamics [193] and cellular communication [194], all processes known to be impaired in AD.

According to the current knowledge regarding OS it is not clear if it OS increase could trigger AD onset. However, increased OS is often observed in the brain of early-stage AD subjects [195]. Further, it seems to have a key role in AD severity and propagation. Indeed, it has been demonstrated that Aβ enhances OS, and represents a source of radical oxygen and nitrogen species (ROS, RNS) [186]. This process is mainly linked with the function of important proteic mediators of OS, including NOX, TGF-β, NF-κB and Nrf2 [196]. The increased OS state in turn triggers several molecular events that are strictly linked with AD symptomatology development [197].

First of all, elevated ROS and RNS concentrations promote Tau phosphorylation, which in turn promotes the destabilization of microtubules. In neurons, microtubules are of primary importance since they maintain these cells polarization and intracellular trafficking [189]. Their destabilization often translates in a decreased function of synapses [198].

ROS can propagate toward the membrane, and then oxidize proteins and nucleic acids, as confirmed by post-mortem AD patients' investigations. In particular, nucleic acid oxidization can cause lethal damage to cell (please refer to Mitochondria section) but can also promote protein aggregation. Indeed, recent observations indicated that Pre-translational mRNA oxidation synthetize peptides that are prone to aggregate [199]. mRNA oxidation seems to be a cytoanatomically selective event that hit mRNAs translated by ribosomes that reside tethered to the mitochondrial outer membrane [32]. The aggregates heavily impair the physiological cell functions. In neurons this could have severe consequences for the normal brain function.

Further, OS exert its effect on the choline recycling from the synapse processes, leading to ACh deficiency [200]. In late stage AD, levels of presynaptic high-affinity choline transporter 1 (CHT1) were observed to be decreased in synaptosomes in the hippocampus and neocortex of humans [201]. Further, high OS inactivates nAChRs, thus inducing long-term depression of cholinergic transmission [202].

An increased OS state leads to an imbalance between pro- and anti-apoptotic processes [153],[203], leading to apoptosis and then neurodegeneration [204]. Interestingly, particularly low ROS concentrations may trigger proliferative signals in neurons, which highly reduce these cells functionality [204].

ROS can also regulate BBB function through enhancing the expression of several metallo-proteinases, and in particular the 9 isoform (MMP9). MMP9 expression is important in brain micro-vascular environment since its alterations are associated with an increased BBB permeability that promotes AD progression through the extravasation of inflammatory factors and ROS in the brain [205],[206].

Another effect of ROS is to influence the energy metabolism of the brain: it indirectly regulates neuronal cells permeability to glucose, decreasing GLUT-3 expression in AD neurons [207],[208] and GLUT-1 in BBB [207]. These molecular events lead to a glucose hypometabolism state in the brain, which has relevant consequences in cells like neurons, whose physiological roles require high levels of energy [186].

Finally, excessive ROS inevitably lead to lipid peroxidation [209]. Polyunsaturated fatty acids are rich in the brain [210]. They could be degraded into malondialdehyde, which causes DNA damage and toxic stress in cells [211].

## 7. Energy metabolism

Numerous evidences implicate Energy metabolism with AD development [212]. In particular, glucose hypometabolism has been detected in the frontal, parietal, temporal, and posterior cingulate cortices of AD patients, and interests especially the basal forebrain cholinergic neurons degeneration [213]. AD intracellular lesions usually develop in neurons presenting long and thin axons compared to the soma [214] and present a reduced or absent myelin sheath. It is interesting to note that these type of neurons are the ones with highest energy requirements [215]. From a molecular point of view, glucose hypometabolism is likely linked with OS. For instance, it was observed that the expression and function of key enzymes in the glycolytic cascade is reduced with high ROS levels [32],[216],[217]. Energy alterations were also linked to a decreased functionality of enzymes related to TCA cycle, which lead neurons to a hypometabolic state [200],[218]–[222]. High Cerebrospinal fluid pyruvate and lactate has been widely reported in AD patients compared with healthy elderly controls [223].

Furthermore, chronic treatment with pyruvate could alleviate short and long-term memory deficits via other pathogenic pathways without reducing amyloid- and Tau-dependent pathology in preclinical AD models [224]. Insulin signaling has been the focus of multiple studies for its association with AD [225]–[228]; Aβ oligomers can bind insulin receptors causing their internalization. The sheer increase of these oligomers in AD subjects triggers an inhibition of the insulin signaling pathways [229]. In sum, the alteration of insulin signaling (or an increased resistance to insulin) ultimately triggers neuroinflammation and neurodegenerative processes through: (1) an increase of Aβ concentrations; and (2) a GSK3β-dependent (Glycogen Synthase Kinase 3β) hyper-phosphorylation of Tau protein [229]–[231]. The potential role of insulin signaling in AD is supported by the observation that insulin-related pathologies, like Diabetes Mellitus, increase AD risk [232].

## 8. Vascular related mechanics

Cerebrovascular abnormalities are common comorbidity in patients with Alzheimer's disease (AD) [233]. They may concur to the onset of cognitive impairment and dementia.

Vascular dysfunctions cause altered brain blood flow and pressure at the level of the brain [234]. These events are detrimental for the normal cerebral function that would result in disturbed homeostasis, but also in blood-brain barrier (BBB) damage and micro-fractures in cerebral vases [235], with increased risk of neuroinflammation [236]. Ultimately, they lead to an increased neuronal death rate and eventually cause the onset and the progression of cognitive impairments [137].

Vascular dysfunctions also set in motion a chain of metabolic events strictly related to AD [237]–[242], including OS. Studies on animal models linked ROS production to the increased concentration levels of the Advanced Glycation Endproducts (AGE) proteins and their receptors (RAGE) in the vascular system. These events are linked to the formation of Aβ plaques [243]–[246]. Interestingly, RAGE is also correlated with inflammatory function and innate immunity, since it has the ability to recognize specific structural motifs (RAGE is also classified as a pattern recognition receptor). A further link with AD events is that brain levels of AGE and RAGE expression have increased in AD subjects [244],[247]. Their expression also tends to increase naturally with age, offering a possible explanation for the increased risk to AD with Age [243],[244],[247].

A chronic hypoperfusion state also promotes the formation of Aβ, through the activation of the adaptive response to hypoxia and reduced clearance via perivascular draining [248],[249]. Consistently, it is interesting to note that Aβ accumulation seems to be mainly localized in brain areas with reduced cerebral blood flow [180]–[182]. Also, the use of angiotensin receptor blockers or angiotensin converting enzyme inhibitors and diuretics has a protective effect against AD [250]–[252]. Nevertheless, the use of these compounds in AD treatment is not indicated, since reduced blood pressure may also trigger detrimental effects including memory impairments and an overall increased risk of AD [253]–[255]. According to our knowledge, in AD the cerebral vascularization undergoes cellular, morphological and structural alterations, which influence the blood flow [235]. However, accumulating evidences suggest that dysfunction of the cerebral vasculature may precede AD neuropathology, implicating a causal role in the etiology of AD, at least in some cases. Indeed, for long time it was believed that some cases of AD may have a vascular origin with neurodegenerative consequences [248]. Whether vascular dysfunctions are causative of AD have yet to be determined. However it is know that the cardiovascular system strictly interacts with AD neuropathology in multiple ways and that the interaction is bidirectional. Making vascular dysfunction an integral part of AD pathophysiology.

## 9. Neurodevelopment and neurotransmission associated processes

Neurodevelopmental and Neurotransmission related pathways are likely associated with AD development and in particular with its cognitive symptoms [256]. Physiologically, these processes consists in the proliferation, differentiation and maturation of neural stem cells (NSC) and the modulation of their interactions through synapse- and neurotransmission-related processes.

Several reports have indicated a significant reduction of 5-HT [257], DA [258] and NE [259] levels as well as their receptors in AD brain. In AD, loss of 5-HT results in depression, anxiety and agitation [260], dysregulation of DA release leads to reward-mediated memory formation deficits [261], and low level of NE impairs spatial memory function [262]. Additionally, the cholinergic system, which regulates memory function and behavior via the release of the neurotransmitter acetylcholine (ACh) [263], was found to be altered in AD. Accumulation of intraneuronal Aβ in AD degenerates basal forebrain cholinergic neurons and reduces ACh levels [264], which in turn leads to memory deficits [265]. Further, mitochondrial dysfunctions are associated with monoaminergic inactivity through various mechanisms. Higher MAO expression, in particular in association with the APOE4, also contributes to a reduced production of monoamines [200].

Further, literature evidences pointed out how pruning pathway becomes aberrantly up-regulated in early stages of AD, mediating synaptic loss [266]. Synaptic pruning is regulated by several signals, including cytokine TGFβ [267], an inflammation-related factor whose levels have been found to be increased in AD [268]–[270].

These processes can be influenced by multiple factors, including OS and inflammation, which were already discussed, and novel processes like epigenetic control [271].

Recently it was observed that some new molecular cascades were triggered by Aβ. Aβ may act through PANX1 expression increase. PANX1 is a protein involved in the modulation of neurotransmission, neurogenesis and synaptic plasticity [189],[272]. An increase of this protein under inflammatory conditions contributes to neuronal death [273]. Aβ can also bind to several postsynaptic partners, including NMDAR and type 1 metabotropic glutamate receptor 5(mGluR5) [189]. The binding of such receptors cause NMDAR-dependent synaptic depression and spine elimination [274]. Recently, the link between Aβ, AD and these processes has been also associated to ryanodine receptors (RyR) function [189].

RyR is Ca^2+^ channel present in three different isoforms (with different responses to Ca^2+^) in the brain. It acts as redox sensors modulating different processes such as neuronal development, apoptosis, gene transcription, synaptic transmission and neuronal plasticity [275]. In particular, Hippocampal RyR has a critical role in many forms of synaptic plasticity [276]. Anomalous RyR channel function occurs in AD pathology [277],[278]. In particular, it was observed that both Aβ and OS would promote aberrant activation of RyR, generating continuous cytoplasmic Ca^2+^ signals [189]. These anomalous Ca^2+^ signals lead to mitochondrial and NOX2-mediated ROS generation [279] and glial activation [280].

Glial activation is an important event in AD. Indeed, recent data demonstrated how the physiological functions of SNC are not linked to neurons functions only. Rather, it was demonstrated that the once “supporting” cells types present in SNC have an important role in the regulation of Brain processes, including neurotransmission and microenvironment homeostasis [165],[166]. As briefly described in Box 2, Microglia modulate synaptogenesis, synapse tagging and elimination or synaptic pruning, help repair damage from injury and modulate synaptic transmission through cytokines [281] and in particular through complement proteins release [271],[282].

These elements act as signals activating circulating macrophages that express complement receptors (C1qR and CR3) and microglial cells in the brain [267],[283],[284]. Microglia activation triggers synapses removal through phagocytosis or by releasing soluble synaptotoxic factors [285]–[287]. Several complement components are normally down-regulated in CNS [266],[283]; their overexpression mediates synapse elimination [266],[267],[283],[288]. Interestingly, studies in human and mouse brains observed an age-related up-regulation of C1q that deposits in synapses, particularly in the hippocampus, one of the most vulnerable regions to synapse loss in AD [289]. C3 has also been shown to contribute to synapse loss and dysfunction in the mouse hippocampus during normal aging [290]. Also, injection of Aβ oligomers in wild-type mice leads to up-regulation of C3 levels, which promote microglial removal of synaptic connections [256]. Further, down-regulating C3 in animal models of AD showed a decreased frequency of neurodegenerative phenomena [291],[292].

Mechanistically, Aβ oligomers increase the expression of C3 in microglia and astrocytes. Elevated C3 levels promote microglia recruitment and mark synapses for elimination. It has to be noted that synaptic loss may be triggered by different mechanisms; some of them might not necessarily follow the trajectory of amyloid buildup [291],[293].

Microglia function could be altered by several processes, including ROS generation and inflammation [279],[280]. In particular, it was observed that glial cells actively release glutamate and ATP in presence of Aβ. This process trigger pro-apoptotic cascades in neurons [294].

## 10. Autophagy impairments

Autophagy is emerging as an important process in the regulation of neuronal and glial cells health in AD. Physiologically, Autophagy is an adaptive process induced under different forms of stress, including nutrient deprivation, hypoxia, and infection [295].

It is a complex process that consists of several sequential steps, sequestration, degradation, and amino acid/peptide generation, mediated by a unique organelle called the autophagosome, a vesicle that contains cellular material targeted to be degraded (macromolecules and organelles) by an intracellular degradation system [296].

Although all cell types have the ability to turn on autophagy, a growing body of studies suggest the importance of this process, specifically, in neurons [297].

Inhibition of autophagy events is causally linked to neurodegeneration, indicating the relevance of autophagy in the neuronal homeostasis regulation [298]. Of note, there is substantial evidence that deregulation of autophagy occurs in AD patients and AD animal models [186]. In this regard, both genetic and environmental factors connect autophagy with AD. For example, Apolipoprotein E4 (apoE4) overexpression potentially causes an Aβ accumulation in lysosomes, leading to neuronal death in the hippocampus [299],[300]. Further, emerging evidence indicates that high ROS levels inhibit the fusion between autophagosomes and lysosomes [186].

Studies on animal models of AD reported that restoring the physiological autophagosomes clearance prevents the manifestation of cognitive symptoms [301].

In more detail, authophagy could be further classified Macroautophagy and Mitophagy: both are somewhat implicated in AD.

Macroautophagy, is tighly regulated degradative pathway that targets cellular macromolecules and eliminates them through proteolytic enzymes in autophagolysosomes [302]–[304]. Some data evidenced how this process is impaired in AD brains [303], where a pathological accumulation of autophagosomes can be observed [305]. In details, AD pathogenesis has been linked to through an impaired merging processes between the autophagosomal-lysosomal system and the consequent reduction/block of protein aggregates turnover [306],[307]. Autophagic vacuoles, isolated from a variety of tissues, were shown to be enriched in APP, gamma-secretase components, PSEN1 and nicastrin, which are required to generate Aβ [308],[309]. Interestingly, studies in animal models evidenced how abnormalities in this process occur early in AD pathogenesis, way before the appearance of neurofibrillary tangles or senile plaques. This indicates that induction of macroautophagy is not a consequence of amyloid deposition [306]. According to the authophagic hypothesis, the block of this process and the consequent accumulation of autophagosomes trigger neuronal degeneration [310] and leads to the release of these vesicles in the extracellular space where they form the characteristic AD plaques [306],[307].

Mitophagy, is the selective autophagic removal of mitochondria. This process is particularly important in neurons, where these organelles play a crucial role in cell survival [311]. In physiological conditions, dysfunctional mitochondria are removed from cytoplasm through mitophagy, an event that in AD disease is suppressed by excessive levels of ROS and Aβ [186].

Generally, initiation of the mitophagy pathway occurs via the relocation of cardiolipin, a diphosphatidylglycerol lipid, from the inner mitochondrial membrane to outer mitochondrial membrane [312]. Evidence has indicated that two proteins PTEN-induced putative kinase1 (PINK1) and parkin can also initiate the mitophagy pathway leading to autophagosome-mediated mitochondrial degradation [313]. In AD, high levels of Aβ and Tau inhibit the expression of PINK1 and parkin, thereby reducing the number of autophagosomes leading to increased dysfunctional lysosomes and the severe disease pathology [312],[314].

## 11. Protein misfolding and misfolded protein clearance

AD research is ever increasing, and everyday new suggestive hypotheses are postulated to tentatively explain that “over 90% of cases” (LOAD) with a cryptic cause. Other than the above cited processes, it has been suggested that AD pathological alterations may be attributed to an incorrect folding of proteins [315]. It has been suggested that pathologically misfolded proteins can influence properly folded proteins to alter their conformation, thus resulting in the propagation of disease [316]. Further, it has been observed that misfolded proteins could also trigger chronic inflammation through different activation pathways [317].

The possibility of propagation of the misfolded proteins between cells is not impossible: there are several processes developed for cell-cell communication, including synaptic transmission, direct communication trough gap junction and paracrine signaling [318] as well as vesicle transporting (see exosomes section), through which a cell to cell protein exchange takes place. From a cytoanatomical point of view protein misfolding is a process that mainly take place in Endoplasmatic reticulum (ER). Physiologically, misfolded proteins in this compartment are eliminated through a fine molecular cascade (ER-associated degradation or ERAD) that recognizes, ubiquitinates, and retrotranslocates misfolded proteins to the cytosolic 26S proteasome [319]. Some studies indicate cross talk between ERAD pathways and OS in AD, where the inhibition of some key enzymes correlates with APP accumulation and Aβ production [186]. Interestingly, Tau accumulation can trigger a cycle where the presence of Tau inhibits ERAD (by binding HRD1): in turn ERAD inhibition leads to an increased Tau aggregation (together with other misfolded proteins) [186]. Misfolded protein continue accumulation ultimately lead to the activation of the Unfolded Protein Response, which trigger apoptosis and, consequently, neurodegeneration [320],[321]. For a more in deep discussion on ERAD cascade please refer to [186].

## 12. Metal hypothesis

Metal ions have a significant role in the brain, since they are required to regulate the neuronal activity in the synapses and many other biological functions [186]. There is increasing evidence suggesting that metal balance impairments, either excess or deficiency of metal ions, are involved in a series of processes that can result in neurodegeneration and cell death [322]. These processes are the hallmarks of various neurodegenerative diseases, including AD [130]. Metals are important elements that effect protein conformation, when their homeostasis is disrupted, several protein misfolding events may appear inside the cell [323]. Regarding AD, it was observed that APP play a crucial role in metal homeostasis and increases metal concentrations when its function is altered [324]. Indeed, their concentrations were significantly increased in AD brain samples [325]. The presence of metal ions (especially for zinc, iron and copper) was detected in Aβ aggregates. In particular, it was observed that the amyloidogenic protein and its precursors have the ability to bind metals [130]. At the same time, bound metals can force proteins to alter their conformations [326], promoting metal-Aβ complexes generation and AD progression [32]. Aβ oligomerization reduces the availability of free metal ions. As Cu and Fe are elements needed for the electron transport, their chelation could inhibit oxidative phosphorylation. (which equals to impaired energy production and ROS production) [204]. Other elements, like Al and Fe, are also important determinants of protein conformation. In particular, Al is noteworthy since is a rather strong inducer of Aβ oligomerization and Al-aggregated Aβ exhibit tight binding to the surface of neurons and a high predisposition to form fibrillar deposits in cellular models [327]. Supporting its role in AD, Al is considered to be an environmental risk factor for the disease [327]. Notably, increased Fe levels accompany initial aggregation and accumulation of Aβ in specific brain regions like hippocampus [328]. Moreover, Fe also promotes Tau aggregation [189],[329].

The role of metals in the brain is not only limited to protein folding processes. Indeed, they also play important roles in SNC physiologic mechanics, including neurotransmitter synthesis, neural information processing, and neuronal myelination [330]. It was demonstrated that deficiency of these metals produces severe adverse effects on central nervous system functions, especially learning and memory, functions intimately linked with AD development. Metal levels increase promote several pro-OS reactions and lead to (1) ROS generation through a Fenton-like mechanism [32],[189],[331],[332] (2) increased ROS lifetime [333] and (3) oxidative phosphorylation decrease in mitochondria [32].

Finally, it was observed that Zn^2+^ and Cu^2+^ ions can be secreted into synaptic clefts [334]–[340] and an excess of these metals triggers a cascade of events that lead to hypoperfusion, thus increasing the risk of neuronal death [130]. While this is not one of the principal biologic processes investigated in AD, the role of metals may be more profound than expected. For a more in depth review regarding the role of metals in AD, please refer to [130],[322].

## 13. A role for exosomes?

The detection of Aβ and Tau in AD subjects' cerebrospinal fluid, evidenced the existence of a mechanism to transport these proteins into the extracellular fluid [341]. While it is possible for cell to reverse their content into the extracellular environment (exocytosis), it has been demonstrated that Tau proteins released into extracellular fluid are cut in their mid-region. This cut prevents Tau aggregation which instead is commonly observed in AD [341]; thus, other transport mechanisms should exist.

Exosomes, are small vesicles on the scale of nanometers, which are widely present in extracellular fluids [341]. Recent data revealed that these vesicles are involved in cell-cell information transfer [341],[342], are released by numerous types of cells [343] and carry genetic material, various bioactive proteins and lipids [342]. In particular, recent studies evidenced that exosomes take part into neuron-glia interaction network and their role likely affect important processes [342]. Given, their function, exosomes may contribute to Aß and Tau propagation in AD brains [344]–[348].

Supporting this possibility, there are some observations reporting that in the early stage of AD, some neurons show morphological changes in their endosome compartment, which seems to increase in size; roughly at the same time Aß levels begin to rise [349],[350]. It was postulated that the intracellular Aß accumulates in endosomes [351]. Noteworthy, these endosomes also contain endocytic multivesicular bodies, whose vesicles are precursors of exosomes [352],[353]. Further, Aβ is also released to the extracellular environment in association with exosomes in cellular models of AD [342],[344]. In humans, Aß plaques contains traces of molecular markers of exosomes (flotillin-1 and Alix proteins) [342] and Aß rich exosomes can be found in the blood of AD subjects [354]. Interestingly, injecting exosomes from AD in the cerebrospinal fluid of animal models can induce AD symptomatology [341]. Further, exosomes from Aβ-treated astrocytes contain the pro-apoptotic PAR-4 protein and these vesicles cause PAR-4 associated apoptosis in naive cultures [355].

Finally, some data have demonstrated that mRNA and miRNA species are present in exosomes. It is still not known if miRNA levels variation is a cause or consequence of the neurodegenerative process. However, several reports evidenced that specific miRNAs, including miR-9, miR-29a/b, miR-107, miR-124, miR-128, miR-132, miR-134, miR-137 and miR-212 levels are altered in AD and are associated with neurodevelopmental processes [342]. Exosomes, by transporting these transcripts, could propagate AD-related damage to other cells.

As an additional effort to shed some light on Alzheimer background we also enriched our literature research with a pathway analysis based on recent genetic findings (see Table 1 and supplementary Table 1) in order to evidence functionally organized networks and related biological processes. Multiple clusters (a total of 6) were evidenced from the initial selection of genes. Each one is linked to different biological processes (Gene Ontology: Biological Processes database—http://geneontology.org/) [356]. The results are reported as supplementary material.

## 14. Mitochondria

Concluding the discussion on the biological processes, we want to focus on a central organelle which may very well occupy a central position in LOAD pathophysiology. Mitochondria are the cellular central point for energy-related processes.

The correlation of Mitochondria with AD is known in literature, supported by both genetic and molecular observations and is believed to occupy a relevant position in the pathogenesis of the disease [204]. A trend of inheritance do exist in AD cases: children of female AD subjects are at higher risk for developing AD than children of male AD subjects [357]. Maternally inherited AD may account for over 20% of all LOAD cases [357], supporting a possible involvement of mtDNA genes [358]–[360]. A number of studies have demonstrated that mitochondrial integrity declines with age, affecting multiple brain functions such as memory, learning and sensory processes [307],[361], which are commonly impaired in AD.

The mitochondrial cascade hypothesis was one of the potential theories advanced to explain AD pathogenesis [200],[204]. According to this hypothesis, polymorphisms within mtDNA and nuclear genes encoding for Electron Transport Chain (ETC) determine the efficiency of mitochondrial energy production as well as the accumulation rate of ROS byproducts. ROS production rate is strictly related to mtDNA alterations.

In particular, ROS accumulation rate is a good indicator of mitochondrial life and his energy production efficacy, since higher is ROS rate, higher is the accumulation of mtDNA damage. The decrease of energy production efficacy is mainly due to alterations of the complexes (I–IV), which are one of the most documented changes in AD [200],[362]. As observed in the previous section, OS also triggers lipid peroxidation and protein oxidation.

Interestingly, some recent data pointed on the regulation of nuclear components through ROS signaling [363]. In details, mitochondrial ROS can upregulate the expression of assembly factors of I, II, III, and IV complexes [364], evidencing the bidirectional talk between mitochondria and nucleus [186]. Additionally, miRNAs seem to be implicated in the regulation of ROS signaling [189].

With the increase of ROS three different responses are triggered: (1) Aβ production from APP, which further increase ROS generation; (2) pro-apoptotic cascade by releasing factor such as cytochrome C; (3) hypoxic signaling cascade, which provides a signal for re-entry into mitotic cycling (destined to fail in neurons) and with resultant aneuploidy, Tau phosphorylation, and neurofibrillary tangle formation [204]. Phosphorylated Tau increases dynamin-related protein 1 (DRP1) levels, which ultimately cause excessive mitochondrial fragmentation [200].

The hypothesis etiologically divides LOAD from EOAD. Focusing on Aβ production, in LOAD this may represent a compensatory event occurring in response to the primary mitochondrial pathology, while in EOAD Aβ production is strictly a toxic phenomenon.

This fact was included in a recent redefinition of the mitochondrial hypothesis which was distinguished into primary and secondary mitochondrial cascade [200], stressing the distinction between mitochondrial dysfunction-induced (primary) and Aβ induced (secondary) AD onset. In the latter the dysfunction is merely an intermediated step.

Either way, Aβ production have harmful effects on mitochondria. According to literature data, Aβ contributes to OS [365],[366]. In addition, Aβ promotes mitochondria fragmentation through S-nitrosylation of Drp1 and consequent augmented GTPase activity [307]. It also inhibits transmembrane translocase on mitochondria through cyclophylin D interactions, increases mitochondrial fission and permeability and downregulates mitochondrial fusion [200].

Mitochondria are essential organelles in every human cell type since play a key role in cell survival or death, regulating both energy metabolism and apoptotic pathways. However, their role in neurons is more than that, since these cells have high energy demands, in particular for neuronal synaptic activity [367] and neurotransmission processes in general [200]. In high OS conditions, mitochondria has a limited serotoninergic efficiency caused by membrane permeabilization and altered serotoninergic metabolism. Monoaminergic activity in general is reduced by mitochondria dysfunction [200]. Finally, some recent literature data indicated that mitochondria actively interact with ER through localized subdomain named mitochondria-associated ER membranes. The activity within these areas is increased in AD cells [368]. Notably, this data may infer to a possible bridge between mitochondria functions and cells protein production, including potential protein misfolding-related processes.

## 15. The impact of the different biological contributors toward the risk

With the exception of around 5% of autosomal dominant AD mutations (EOAD), a number of other biological processes contribute to AD cases world-wide. As introduced in the previous sections it is unlikely that a single factor (or mechanics) is responsible for synaptic dysfunctions and the other pathological changes that occur in AD subjects' brain. In this paragraph, we want to briefly illustrates how the several pathways discussed, may be linked through transduction signaling cascades. While the latter may be not directly causative of AD, they undoubtedly have a key role in AD propagation and symptoms manifestation.

As discussed in the Mitochondria section, whether Aβ production is the causative event (EOAD) or caused by other processes, including mitochondria dysfunction, its cytoxicity is primarily exerted on mitochondria. The two main consequences are an increased OS and a reduced (aerobic) energy production. ROS acts on lipids promoting their peroxidation and perturbing their homeostasis. Physiologically, the latter are used not only for energy production but also as secondary messengers [369]. Several genes involved in lipid homeostasis, including APOE, CLU, SORL1 and ABCA7 were correlated with AD [370]. Polyunsaturated fatty acids could participate in myriad signal transduction within the brain directly or after enzymatic conversion to a series of mediators. For example, Activated sphingolipids are signaling molecules that serve as intracellular second messengers [371]. Their shortage has a potentiation effect on Aβ secretion [342], thus causing its accumulation (and plaques formation). Literature data indicates that this shortage is often observed in AD brains, especially in the inferior temporal and middle frontal gyri [369]. Further, Cholesterol and its esters low concentration correlate with increased of Aβ production [370].

Cellular Ca^2+^ signals regulate several important processes in neurons [372],[373]. Literature evidences indicate that its dyshomeostasis may play a key role in the pathogenesis of AD [374],[375]. Interestingly, there is ample some data reports that Ca^2+^ dysregulation may even precede the formation of Aβ plaques and neurofibrillary tangles in AD brain, suggesting disruption in cytoplasmic Ca^2+^ is an earlier event [372],[376].

Intracellular Ca^2+^ is usually stored in the ER. Its release in the cytosol is controlled by RyRs and inositol 1,4,5-trisphosphate receptors (InsP_3_R) [377]. Ca^2+^ intake from extracellular compartment is controlled by store-operated Ca^2+^ entry (SOCE) pathway and, by voltage-gated Ca^2+^ channels (VGCC) in neurons. Also, it has been shown Ca^2+^ influx through the CALHM1 channel or NMDAR stimulates α-secretase processing of APP [375], thus protecting from Aβ accumulation. It was observed that high OS state and Aβ aggregates can interfere with Ca^2+^ homeostasis as they can trigger Ca^2+^ release from ER stores through the InsP_3_R and RyR [375],[378]. Increased Ca^2+^ levels in the cells interferes with the physiological function of VGCCs, thus impairing neurotransmission [373]. Imbalanced cellular Ca^2+^ also contributes to pathophysiological conditions such as accumulation of Aβ plaques and neurofibrillary tangles, protein misfolding, necrosis, apoptosis, autophagy deficits, and degeneration [375],[379]–[381]. Finally, excess cytosolic Ca^2+^ exacerbates mitochondria dysfunction and dysregulates KIF5-Miro-Trak-mediated mitochondrial transport to synapses [200]. For a more detailed discussion on Ca^2+^ please refer to [375],[381].

G protein–coupled receptors (GPCRs) regulate cell responses to external molecules, like neurotransmitters [382],[383] and it was demonstrated that their action is involved in both long- and short-term memory biological processes [384], known to be impaired in AD.

GPCRs can control genetic expression through their interaction with the extracellular signal-regulated kinases (ERKs or ERK1/2). ERKs is involved in a wide array of signals and signal transduction pathways, including the ones involved in the formation of memory traces [385],[386] and the ones involved in the regulation of neuronal plasticity (both protein synthesis and protein phosphorylation in dendrites) [387],[388]. Interestingly, G-GPCRs produce the secondary messenger inositol 1,4,5-triphosphate (IP3) which binds to IP3R on ER and efflux the Ca^2+^ into cytoplasm [381].

Dysfunction of the mammalian target of rapamycin (mTOR) pathway has been implicated in both neurodevelopmental and neurodegenerative diseases, including AD [389]–[392]. Numerous studies have demonstrated mTOR hyperactivation in AD brain [393]. Physiologically, mTOR is a regulator of survival, differentiation, and development of neurons [394]. It was observed that Aβ accumulation activates mTOR pathway through phosphorylation of mTOR inhibitor PRAS40 [390]. Further, hypoenergetic states seems also to trigger this pathway [395]. The aberrant activation of the mTOR pathway leads to an alteration of protein synthesis and autophagy's mechanisms which in turn are related to the accumulation of inclusion bodies, a common feature of AD ([391],[392],[395] and references therein). During neural development, mTOR regulates neuronal growth, differentiation and interconnectivity [396]–[398]. It regulates homeostasis by directly influencing gene transcription, protein and lipid synthesis and organelle biogenesis and maintenance [391]. This pathway appears to be influenced by ERKs and GPCRs action [399]. In turn mTOR pathway inhibits PI3K/Akt signaling, thus triggering GSK-3β activation [394]. Accumulating evidence also supports a correlation between alterations in mTOR signaling and aging [400]. Finally, cell cultures from animal models of AD interestingly display a reduced mTOR signaling [401]. Alterations of this pathway may be linked to the excessive burden of misfolded proteins [399].

The balance of pro- and anti-apoptotic Bcl-2 family proteins (i.e. Bcl-2, Bad, Bim and Bax) has been known to have a role in neuronal cell death [402]. The levels of these proteins are altered in AD brains neurons [402]. Although the exact molecular details are not completely described, it is believed that Aβ is able to increase Bim and decrease Bcl-2 levels [402]. The pro-apoptotic protein (Bim) and anti-apoptotic protein (Bcl-2) are known to exert their activity through heterodimerizxation with Bax protein [403]. Bax is usually localized in the cytosol or associated with the membranes of mitochondria and/or ER [404]. Upon activation, Bax changes conformation and dwells into mitochondrial membranes where it promotes the release of cytochrome C and triggers apoptosis [405],[406]. With the presence Aβ, Bim overexpression leads to the activation of Bax which triggers neuronal cell death [402]. The activation of this cascade is strictly correlated with the neurodegenerative events associated with AD.

The increase of Aβ production negatively impact survivability of neurons. In physiological conditions, it has been observed that the predominant APP-cleaved fragment (sAPPα) interacts with IGF1 and insulin receptor. This interaction together with different neurotropins (NGF, BDNF) mediates the activation of the PI3K/Akt pathway. This action promotes neuronal survival, possibly through phosphorylation and inhibition of glycogen synthase kinase-3β (GSK-3β) [395]. The accumulation of Aβ directly compete with the effects mediated by the different neurotropins, downregulating PI3K/Akt pathway and ultimately overactivating GSK-3β [395]. The activation of GSK-3β accounts for several features of AD such as increased Aβ production and plaque accumulation [407], reduced memory performance, neurogenesis and synaptogenesis (astroglial-related) [395],[408], as well as increased Tau phosphorylation, Inflammation (microglia) and neurodegeneration (mitochondrial intrinsic apoptotic and the death receptor-mediated extrinsic apoptotic pathways) [409]. Additionally, Aβ direct competition with insulin for its receptor, increases the latter levels in the brain microenvironment. High insulin levels promote inflammatory responses in the brain, based on increased TNFα, interleukin 1β and 6 (IL1β and IL6) [395].

Following the progression of AD, brain neurons find themselves in a status of reduced energy production and the microenvironment has elevated pro-inflammatory cytokines (see previous sections). In these conditions Notch signaling is upregulated [410]. Physiologically, Notch signaling has an essential role in vascular development and (neuro) angiogenesis through the modulation of VEGFR2 [411]. It is also associated with synaptic plasticity, and glial cell activation [412],[413]. Recently, some data hypothesized that Notch signaling may be implicated AD as Notch1 is often found in Aβ plaques [410],[414].

Despite its physiological role, it has been observed that chronic activation of Notch1 negatively impact the brain microenvironment, in particular the delicate connection of the brain with cardiovascular system. Indeed, Notch signaling, in association with VEGF, has been demonstrated to cause impaired blood flow, further reducing the nutrients intake by neurons (worsening the already weak energetic state). Notch also induce BBB leakages which has severe impact on the brain and may accelerate Aβ accumulation [410]. Finally, Notch is involved in regulating APP proteolysis [410]. Despite the still lacking data, Notch signaling, is a potential candidate that links various AD related processes with vascular impairments and may explain how they interact.

Aβ accumulation in AD is strictly linked with the upregulation of DKK1 gene [415],[416]. The translated protein is an inhibitor of Wnt (canonical) pathway. Physiologically, Wnt signaling has important functions linked to plasticity and cognition. Among these, it promotes the endocytosis, the recycle of vesicles and modulate neurotransmitter release [417] and receptor transcription [418]. It also facilitates the induction of LTP [419].

**Figure 1.**
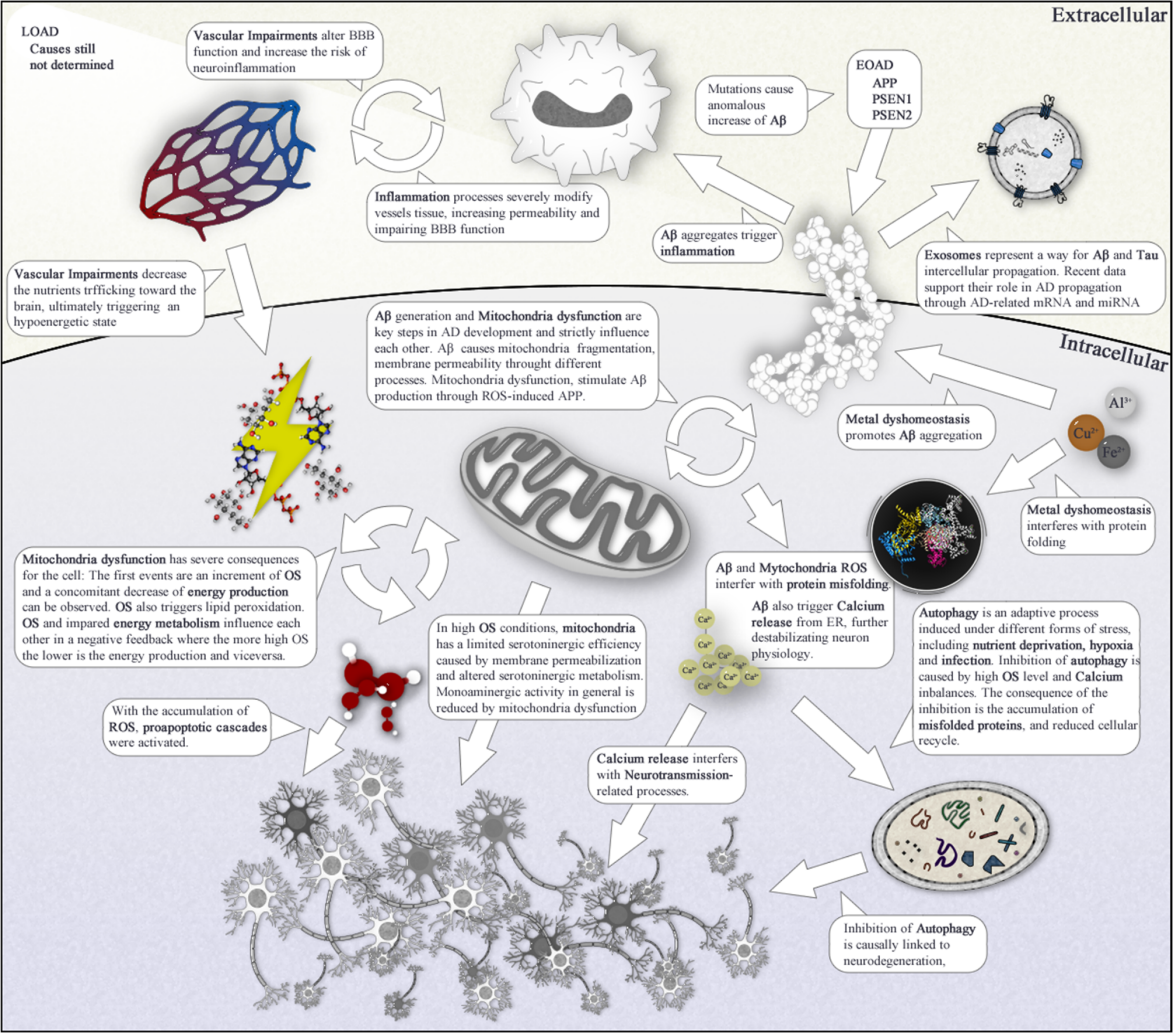
Graphical view of pathways connections.

Wnt signaling has three major cascades: the canonical Wnt/ß-catenin, planar cell polarity (PCP), and Wnt/Ca^2+^ pathways. Interestingly, Wnt/ß-catenin and Wnt/PCP modulate synapse maturation; specifically, Wnt/ß-catenin promotes synapses stability, while Wnt/PCP promotes synapse retraction [420]. With, the over expression of DKK1 by Aβ, the canonical pathway is suppressed skewing the balance toward the non-canonical PCP pathway. This event accelerates synaptic damage [420] and participate to the progression of cognitive symptoms of AD. Further, canonical Wnt/ß-catenin pathway promotes the phosphorylation (and inactivation) of GSK-3β [419]. Thus, its inhibition produces similar effects to the ones described in PI3K/Akt signaling. The diagram in Figure 1 reports a simplified view of the pathways connections discussed.

## 16. Methods

The main aim of literature research was to extrapolate biological pathways potentially involved with the etiopathogenesis of Alzheimer disease. Phase (1) A preliminary research was done selecting reviews (from 2013 to 2018) on PubMed using the keywords: Alzheimer AND (Genetics OR Biological cascades) in order to prepare a background for the paper. At the time of the research, 1427 elements were obtained. Data were screened for genes association and molecular cascades. Phase (2) Genes with multiple references of association with AD plus the ones extracted from Alzgene.org dataset were grouped and clustered (using Gene Ontology Database for gene-gene correlations). Cluster data from phase 2 was also used to perform pathway analyses (see exploratory data for details, and Supplementary Figure 1 for a summary). The data obtained was used as criteria for pathway selection. Phase (3) Data from phase 1 and phase 2 were used to a more detailed research: each pathway was used as keyword (Alzheimer AND Pathway name) for a refined literature research on PubMed and Google scholar. A total of 2204 (Immune System), 3836 (Inflammation), 4653 (Oxidative Stress), 2792 (Energy Metabolism), 746 (Autophagy), 130 (Exosomes-mechanics), 469 (Vascular-Related Mechanics), 4150 (Signal Transduction) and 169 (Neurodevelopment and Neurotransmission) works, partially overlapping, were obtained. Starting from the most recent ones, literature findings were filtered and screened for information.

## 17. Conclusive remarks

AD is one of the principal causes of disability and decreased quality of life among the elderly. Despite the active research on the field, many fundamental questions remain regarding the molecular background of this disease.

The old theories linking AD to the genes directly related to Aβ formation and Tau hyper-phosphorylation cannot explain the complexity of AD and neither give a definite target for treatment. Indeed, the conventional treatments based on the Aβ hypothesis have failed to treat or prevent AD. Around 99.6% of drug candidates targeting amyloid pathways, including β secretase inhibitors, γ secretase inhibitors, and Aβ itself, do not have a significant therapeutic effect on AD subjects [9].

Recent studies and the new hypotheses for AD have indicated many new potential targets [421]–[424] even though we are still far from a definitive solution. The evidences derived from the study of the disease and its treatment, together with the current concept of AD as “multifactorial pathology”, clearly indicates the necessity to consider wider approaches which include the complex interactions between different pathways. Part of these biological processes was introduced and described in this review.

Other than the familial form-associated genes (PSEN1, PSEN2, APP) and the high risk variants of APOe, the AD background is obscure. Its complexity is not to be underestimated and it is likely that multiple factor may trigger AD pathological changes in the brain. From what it was discussed, whether the etiological cause, AD progression hit Mitochondria at some point. The central role of this organelle is plausible, considering its importance in neurons. It's destabilization could arise following Aβ accumulation (EOAD main etiological cause), reduced intake of nutrients (which makes mitochondria sensible to vascular impairments). Even aging itself (accumulation of damage on mtDNA) slowly decrease mitochondria efficiency. However, mitochondria dysfunction alone is not enough to explain why the damage is mainly restricted on neurons. Additionally, all the propagation mechanics remain elusive and the molecular cascades behind the propagation path (see Box 1) is not completely understood.

The key to further deepen the studies of AD understands how these processes interact with each other. Thereby, a focus on multiple pathways or functional cascades may be useful to pinpoint novel potential target for AD treatment.
