# Supplementary material for: The biological pathways of Alzheimer disease: a review
Source: AIMS Neurosci. 2020 Dec 16;8(1):86–132. doi: 10.3934/Neuroscience.2021005 (PMC7815481; doi:10.3934/Neuroscience.2021005)
Supplement: Supplementary file 1 [file neurosci-08-01-005-s001.pdf]

Neuroscience

AIMS Neuroscience, 8(1): 86–132.
DOI: 10.3934/Neuroscience.2021005
Received: 24 August 2020
Accepted: 23 November 2020
Published: 16 December 2020

http://www.aimspress.com/journal/neuroscience

Review

The biological pathways of Alzheimer disease: a review

Marco Calabrò, Carmela Rinaldi, Giuseppe Santoro and Concetta Crisafulli*

Department of Biomedical and Dental Sciences and Morphofunctional Imaging, University of Messina, Italy

* **Correspondence:** Email: ccrisafulli@unime.it; Tel: +39 0902213373; Fax: +39 090692449.

Supplementary material

Supplementary Box 1. Pharmacological Treatment

Two categories of drugs are presently used for AD treatment. The Cholinesterase Inhibitors (ChEI), act by increasing the level of acetylcholine, a neurotransmitter important for learning, memory and cognitive functions [1,2]. The latter is designed to block NMDA receptors and to slow down intracellular Ca^{2+} accumulation.

ChEIs

Tacrine: is the first ChEI to be approved specifically for the symptomatic treatment AD (mild to moderate). Tacrine present some adverse effects, in particular related to hepatotoxicity [3] and thus requires baseline and multiple follow-up liver enzyme determinations. This, along with its mutidose administration (10 mg 4 times daily), make it not the first choice for treatment, and limit its use to subjects not tolerating or not responding to other ChEIs. Regarding its pharmacokinetics properties, Tacrine is metabolized by the liver via the cytochrome P450 1A2 isoenzyme system. Therefore, potential interaction with other compounds metabolized by this isoenzymes should be considered. Tacrine should be avoided in patients with liver disease.

Donepezil: is the second ChEI approved for symptomatic treatment of mild to moderate AD [4]. Some adverse effects are present, in particular related to interaction with other compounds, like paroxetine, which increase the frequency of confusion and agitation states in treated AD subjects [5]. Donepezil is largely metabolized by the liver, although some of the dose is recovered in the urine as unchanged drug (11–17%) [6]. It is metabolized by the cytochrome P450 isoenzymes 2D6 and 3A4. Donepezil should be used with caution in subjects severe hepatic or renal disease.

Rivastigmine: This was the third ChEI approved by the FDA for symptomatic treatment of mild to moderate AD in the United States. Rivastigmine should be titrated every 4 weeks, as opposed to every 2 weeks, as recommended when the drug was first made available. Slower titration and taking rivastigmine with a full meal significantly improves tolerability, especially with regard to gastrointestinal side effects. One unique feature of rivastigmine that distinguishes it from other ChEIs is the very low risk of drug interactions in AD patients receiving multiple medications for ‘‘real-world’’ comorbidities [7]. This is because the metabolism of rivastigmine occurs primarily via enzymatic cleavage (hydrolysis) by cholinesterases at the site of action and does not require the cytochrome P450 enzyme system. The starting dose is 1.5 mg twice a day with meals (breakfast and supper), and this dose is increased by 3 mg/day, not faster than every 4 weeks (as tolerated), to a therapeutic dose of 6–12 mg/day. The highest tolerated dose is recommended, as there is some evidence that higher doses may provide greater benefits. A more rapid progression of AD while receiving placebo treatment was predictive of a significantly stronger patient response to rivastigmine therapy on various measures [8]. Laboratory monitoring is not required.

Continued on next page

Supplementary Box 1. Pharmacological Treatment

Galantamine: This was the fourth ChEI approved by the FDA for symptomatic treatment of mild to moderate AD in the United States. Metabolism is hepatic via glucuronidation and the cytochrome P450 isoenzymes 2D6 and 3A4; interactions with other drugs that are metabolized through this pathway are therefore possible. Caution should be used in patients with liver disease. The starting dose is 4 mg twice a day, and this dose is increased every 4 weeks. The therapeutic dose is 16–24 mg/day. A 6-month study showed no additional benefit and a higher rate of side effects with a dose of 32 mg/day [9]. Laboratory monitoring is not required.

NMDA Blockers

Memantine: a noncompetitive, highly voltage- dependent NMDA antagonist, has been approved for use in the treatment of dementia in Germany for over 10 years and recently was approved for use in the treatment of AD in the European Union. It has been found to be useful in more advanced (moderate to severe) cases of AD [10]. Patients with AD in the United States may import the drug or consider participating in double-blind, placebo-controlled studies currently underway at numerous sites all over the country. The FDA is currently reviewing the data for approval of its use in the United States. The recommended starting dose is 5 mg daily, which is increased to 10 mg daily after 4–6 weeks. Morning dosing of donepezil may be preferable in some patients who experience nightmares or insomnia. It may be taken without regard to meals unless gastrointestinal side effects occur, in which case it should be taken with meals. Although there is 1 case report of fulminant hepatitis with the concomitant use of donepezil and sertraline [11], laboratory monitoring of liver enzymes is not required.

Other compounds are also used (with less frequency) for AD treatment [2]. Unfortunately, none of them represent a definitive solution to the pathology.

Supplementary Table 1. Genetic associations with Alzheimer's Disease in recent publications.

Literature Data	Identified Associations	
Known familiar genes [12–14]		
	Dominant	Risk
	APP, PSEN1, PSEN2	APOE
[15]		
	Significant (after statistical correction)	Trend
	ABCA7, AC099552.4, BCAM, CBLC, GAS2L2, IGH3, MS4A6A, PILRA, STAG3, TREM2, ZNF655	CBX3, DTNBP1, HLA-DPA1, LDB3, MUC4, NSF, OPRL1, RHBDD1, RPS16, SIRPB1
[16]		
	Biomarkers	
	APLP2, APOC1, ATAD1, BDNF, BMPR1A, CAPN2, CARD8, CTSS, GFRA2, GLRX3, GSN, HMGCS1, IGF1, ITPRIP, MAPJ1, MAPK10, MAPK8, PADI2, PCSK1, PFKFB3, PON2, PPP2R2B, PPP3CB, RBP4, RFC3, SEBPD, SERPINF1, SLC27A2, SMC3, STAMBPL1, TF, TIMP1, TMED10, TNFRSF1A, VLDLR	
[17]		
	Potential AD Drug Targets	
	A2M, ADAM12, APOD, C1QB, CD86, DNNP, GABRG2, HLA-B, HSPG2, IL-10, LEP, MTTP, NEK4, NOS1AP, PIK3C2A, RELN, TARBP1, TF	
[18]		
	Novel associations	Known AD candidate genes
	AC074212.3, ADAM10, ADAMTS4, ALPK2, APH1B, CLNK, CNTNAP2, HESX1, HKAT8	ABCA7, ABI3, BIN1, BZRAP2-AS1, CASS4, CD2AP, CD33, CLU, CR1, ECHDC3, EPHA1, HLA-DRB1, HS3ST1, INPPD5, MS4A6A, PICALM, PTK2B, SCIMP, SLC24A4, SORL1, SUZ12P1, TREM2, ZCWPW1

Continued on next page

Literature Data	Identified Associations	
[19]		
	Significant associations	Known AD candidate genes
	FERMT2, PICALM, SLC24A4	ABCA7, AKAP9, BIN1, CASS4, CD2AP, CD33, CELF1, CLU, CR1, EPHA1, GRN, HLA-DRB1, INPP5D, MAPT, MEF2C, MS4A6A, NME8, PLD3, PTK2B, RIN3, SORL1, TREM2, TREML2, ZCWPW1
[20]		
	Associated genes	
	ABCA7, AKAP9, ADAM10, BIN1, CASS4, CD2AP, CD33, CELF1, CLU, CR1, DSG2, EPHA1, FERMT2, HLA-DRB5/DRB1, INPP5D, MEF2C, MS4A6A, NME8, PICALM, PLD3, PTK2B, RIN3, SLC24A4, SORL1, TREM2, UNC5C, ZCWPW1	
[21]		
	Associated genes	
	SPI1	

Cluster 1. Informative Data.

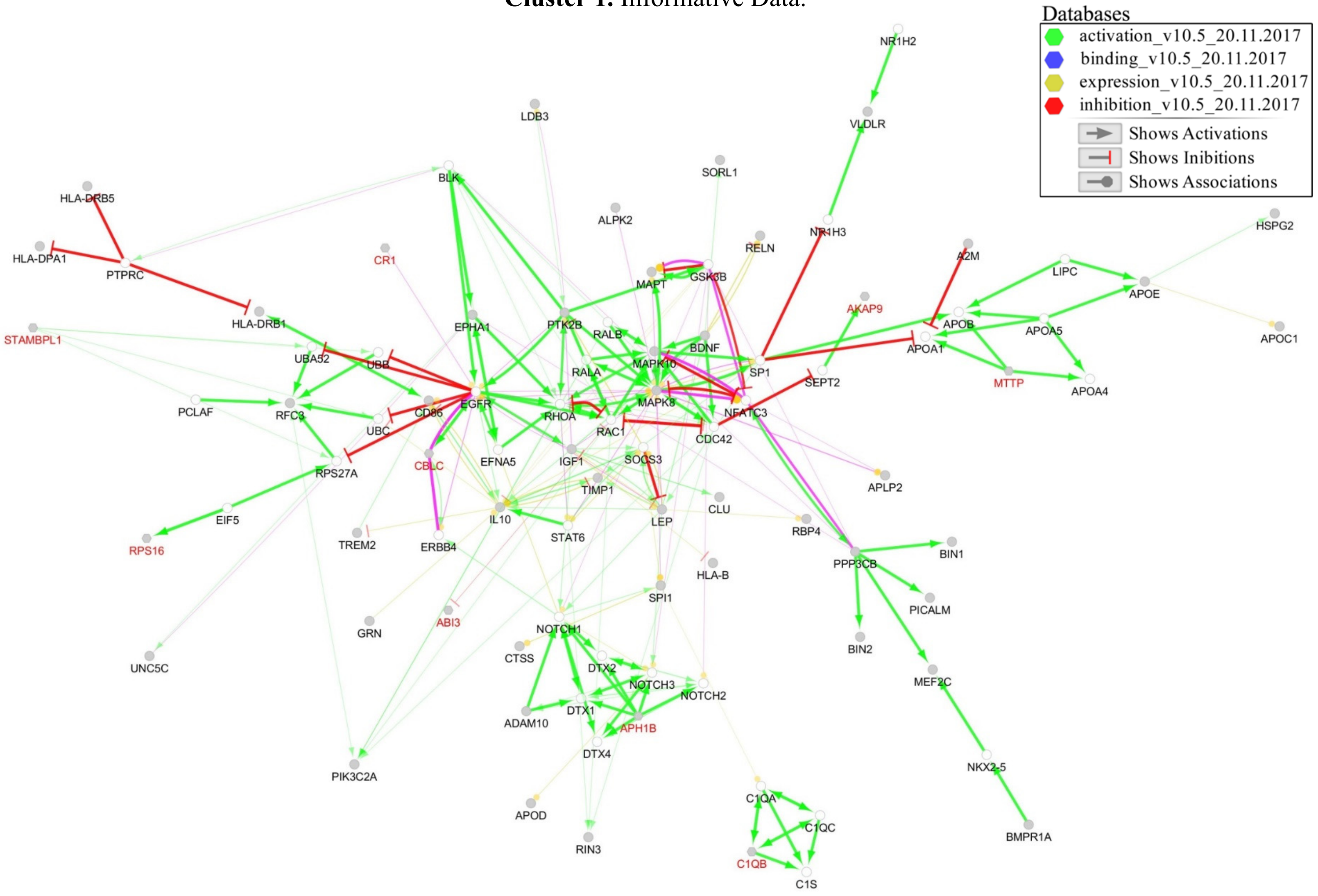

Cluster 1. Informative Data.

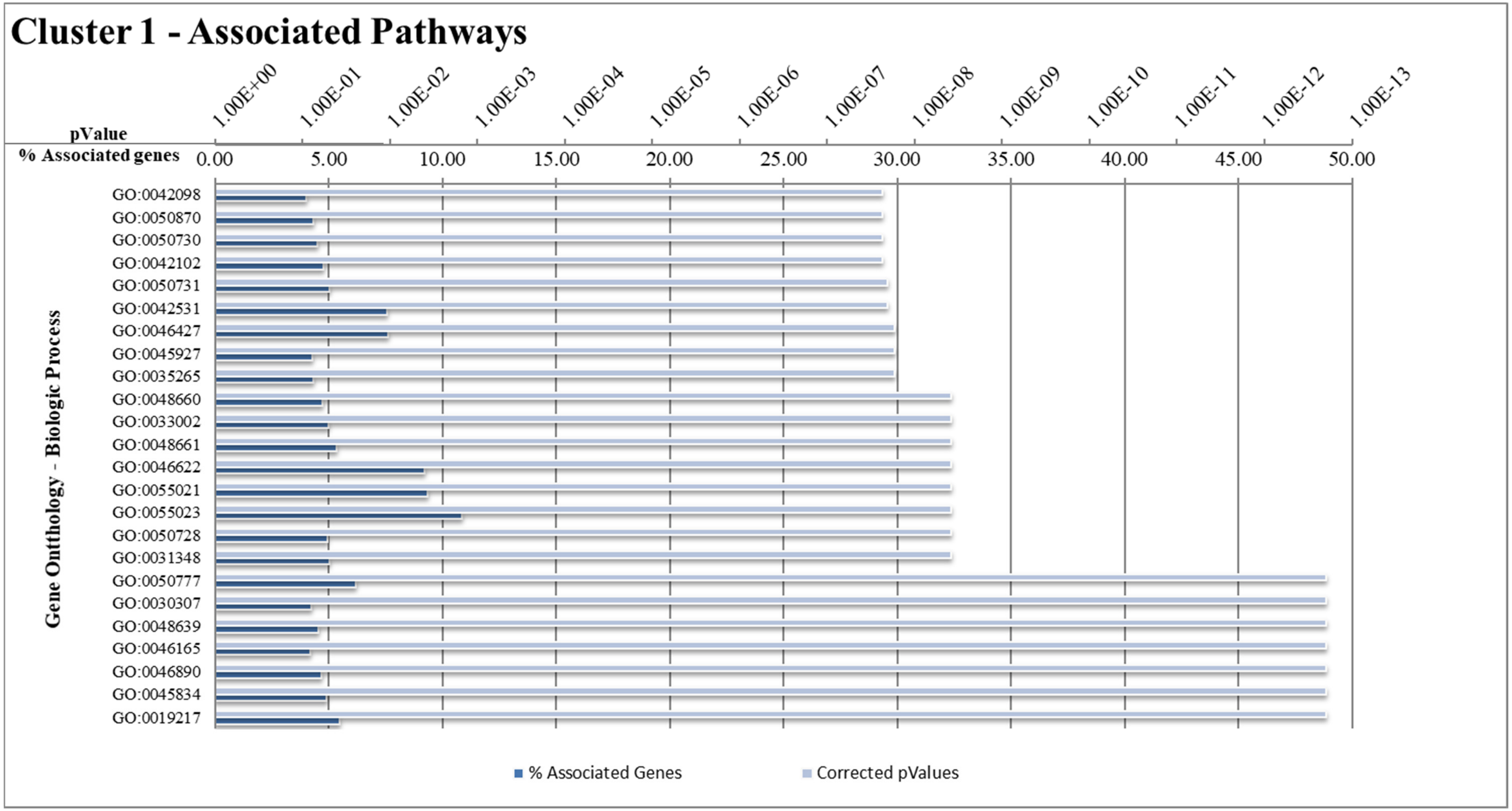

Cluster 1. Informative Data.

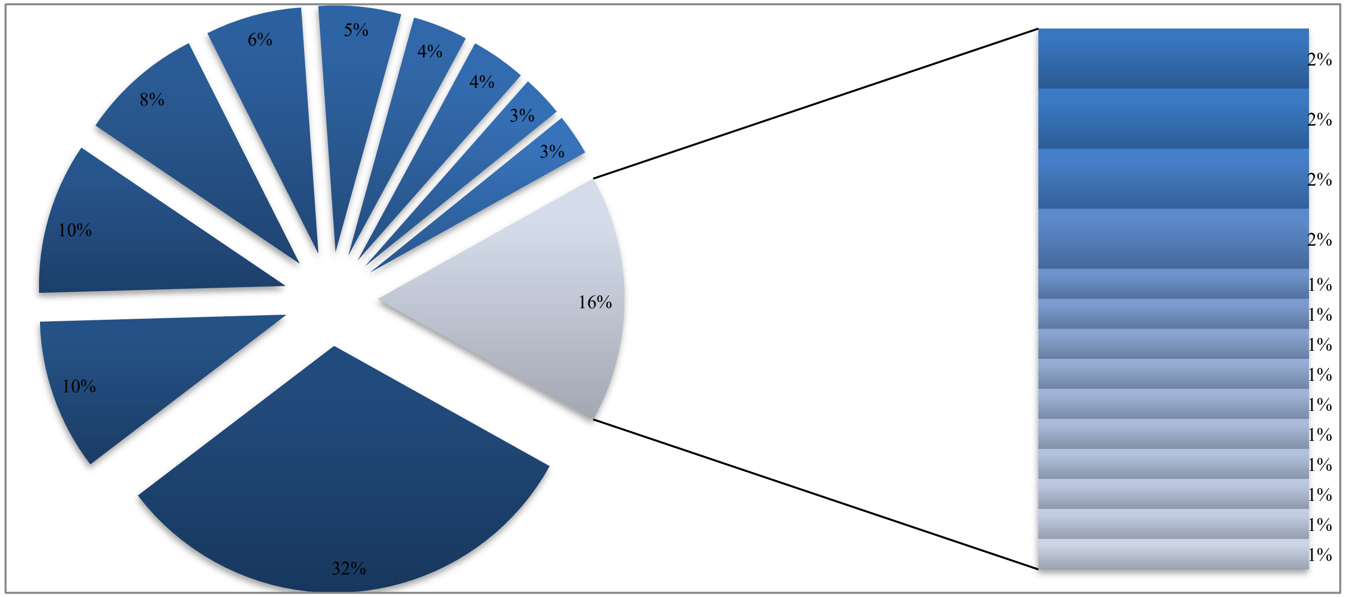

- 31.53% cholesterol transport
- 9.91% protein-lipid complex subunit organization
- 9.91% positive regulation of supramolecular fiber organization
- 8.11% muscle cell proliferation
- 6.31% regulation of peptidyl-tyrosine phosphorylation
- 5.41% regulation of transcription from RNA polymerase II promoter in response to hypoxia
- 3.60% regulation of cell size
- 3.60% blood vessel endothelial cell migration
- 2.70% positive regulation of growth
- 2.70% negative regulation of immune response
- 1.80% glial cell differentiation
- 1.80% Notch signaling pathway
- 1.80% regulation of nitric-oxide synthase activity
- 1.80% negative regulation of amyloid precursor protein catabolic process
- 0.90% tissue regeneration
- 0.90% regulation of humoral immune response
- 0.90% animal organ regeneration
- 0.90% regeneration
- 0.90% positive regulation of small GTPase mediated signal transduction
- 0.90% artery morphogenesis
- 0.90% positive regulation of hormone secretion
- 0.90% negative regulation of innate immune response
- 0.90% cardiac septum development
- 0.90% vasoconstriction

Cluster 1. Informative Data.

Gene Ontology Identification Number		Raw pValue		Corrected pValue*		% Associated Genes	Nr. Genes
		Term	Group	Term	Group		
GO:0042098	T cell proliferation	5.31E-07	8.28E-15	2.55E-05	1.99E-13	4.02	10.00
GO:0050870	positive regulation of T cell activation	6.43E-08	8.28E-15	4.05E-06	1.99E-13	4.33	11.00
GO:0050730	regulation of peptidyl-tyrosine phosphorylation	1.15E-10	8.28E-15	1.00E-08	1.99E-13	4.52	15.00
GO:0042102	positive regulation of T cell proliferation	4.36E-05	8.28E-15	3.49E-04	1.99E-13	4.76	6.00
GO:0050731	positive regulation of peptidyl-tyrosine phosphorylation	2.96E-09	8.28E-15	2.25E-07	1.99E-13	5.02	12.00
GO:0042531	positive regulation of tyrosine phosphorylation of STAT protein	2.98E-06	8.28E-15	1.01E-04	1.99E-13	7.59	6.00
GO:0046427	positive regulation of JAK-STAT cascade	5.98E-08	8.28E-15	3.83E-06	1.99E-13	7.62	8.00
GO:0045927	positive regulation of growth	9.82E-10	1.66E-10	8.06E-08	3.82E-09	4.29	14.00
GO:0035265	organ growth	1.05E-06	1.66E-10	4.43E-05	3.82E-09	4.35	9.00
GO:0048660	regulation of smooth muscle cell proliferation	2.30E-06	1.66E-10	8.50E-05	3.82E-09	4.73	8.00
GO:0033002	muscle cell proliferation	3.25E-09	1.66E-10	2.40E-07	3.82E-09	4.98	12.00
GO:0048661	positive regulation of smooth muscle cell proliferation	2.24E-05	1.66E-10	3.36E-04	3.82E-09	5.36	6.00
GO:0046622	positive regulation of organ growth	9.36E-07	1.66E-10	4.12E-05	3.82E-09	9.23	6.00
GO:0055021	regulation of cardiac muscle tissue growth	1.03E-07	1.66E-10	5.84E-06	3.82E-09	9.33	7.00
GO:0055023	positive regulation of cardiac muscle tissue growth	3.50E-06	1.66E-10	1.05E-04	3.82E-09	10.87	5.00

Continued on next page

Gene Ontology Identification Number		Raw pValue		Corrected pValue*		% Associated Genes	Nr. Genes
		Term	Group	Term	Group		
GO:0050728	negative regulation of inflammatory response	1.67E-06	7.72E-10	6.53E-05	1.70E-08	4.94	8.00
GO:0031348	negative regulation of defense response	2.96E-09	7.72E-10	2.25E-07	1.70E-08	5.02	12.00
GO:0050777	negative regulation of immune response	1.60E-09	7.72E-10	1.25E-07	1.70E-08	6.18	11.00
GO:0030307	positive regulation of cell growth	1.29E-06	9.82E-10	5.14E-05	2.06E-08	4.25	9.00
GO:0048639	positive regulation of developmental growth	1.62E-07	9.82E-10	8.77E-06	2.06E-08	4.57	10.00
GO:0046165	alcohol biosynthetic process	5.48E-06	1.18E-09	1.43E-04	2.36E-08	4.21	8.00
GO:0046890	regulation of lipid biosynthetic process	2.91E-08	1.18E-09	2.00E-06	2.36E-08	4.68	11.00
GO:0045834	positive regulation of lipid metabolic process	3.93E-07	1.18E-09	1.96E-05	2.36E-08	4.89	9.00
GO:0019217	regulation of fatty acid metabolic process	3.93E-06	1.18E-09	1.10E-04	2.36E-08	5.47	7.00
GO:0019218	regulation of steroid metabolic process	6.51E-07	1.18E-09	3.00E-05	2.36E-08	5.59	8.00
GO:0022600	digestive system process	3.01E-06	1.18E-09	9.94E-05	2.36E-08	5.69	7.00
GO:0008203	cholesterol metabolic process	3.01E-09	1.18E-09	2.26E-07	2.36E-08	5.82	11.00
GO:1905952	regulation of lipid localization	9.92E-09	1.18E-09	7.05E-07	2.36E-08	6.13	10.00
GO:0046889	positive regulation of lipid biosynthetic process	9.74E-07	1.18E-09	4.19E-05	2.36E-08	6.73	7.00
GO:0031102	neuron projection regeneration	2.10E-05	1.18E-09	3.37E-04	2.36E-08	7.58	5.00
GO:0034381	plasma lipoprotein particle clearance	2.37E-06	1.18E-09	8.53E-05	2.36E-08	7.89	6.00
GO:0060193	positive regulation of lipase activity	3.47E-08	1.18E-09	2.36E-06	2.36E-08	8.16	8.00

Continued on next page

Gene Ontology Identification Number		Raw pValue		Corrected pValue*		% Associated Genes	Nr. Genes
		Term	Group	Term	Group		
GO:0060191	regulation of lipase activity	5.45E-11	1.18E-09	4.85E-09	2.36E-08	8.46	11.00
GO:1905953	negative regulation of lipid localization	9.36E-06	1.18E-09	2.15E-04	2.36E-08	8.93	5.00
GO:0030301	cholesterol transport	1.56E-11	1.18E-09	1.41E-09	2.36E-08	9.48	11.00
GO:0070328	triglyceride homeostasis	1.69E-07	1.18E-09	8.96E-06	2.36E-08	12.24	6.00
GO:0033344	cholesterol efflux	8.66E-10	1.18E-09	7.18E-08	2.36E-08	12.90	8.00
GO:0090207	regulation of triglyceride metabolic process	7.56E-08	1.18E-09	4.46E-06	2.36E-08	13.95	6.00
GO:0032374	regulation of cholesterol transport	4.32E-10	1.18E-09	3.71E-08	2.36E-08	14.04	8.00
GO:0034375	high-density lipoprotein particle remodeling	7.39E-08	1.18E-09	4.44E-06	2.36E-08	22.73	5.00
GO:0051004	regulation of lipoprotein lipase activity	7.42E-11	1.18E-09	6.53E-09	2.36E-08	25.00	7.00
GO:0043691	reverse cholesterol transport	1.80E-09	1.18E-09	1.39E-07	2.36E-08	25.00	6.00
GO:0034380	high-density lipoprotein particle assembly	4.39E-08	1.18E-09	2.90E-06	2.36E-08	25.00	5.00
GO:0051006	positive regulation of lipoprotein lipase activity	8.67E-09	1.18E-09	6.24E-07	2.36E-08	33.33	5.00
GO:0010873	positive regulation of cholesterol esterification	1.36E-09	1.18E-09	1.07E-07	2.36E-08	45.45	5.00
GO:0010810	regulation of cell-substrate adhesion	1.24E-07	3.89E-09	6.95E-06	7.40E-08	4.06	11.00
GO:0031032	actomyosin structure organization	3.38E-07	3.89E-09	1.72E-05	7.40E-08	4.22	10.00
GO:0010811	positive regulation of cell-substrate adhesion	1.27E-05	3.89E-09	2.54E-04	7.40E-08	4.58	7.00
GO:1902905	positive regulation of supramolecular fiber organization	7.96E-09	3.89E-09	5.81E-07	7.40E-08	4.60	12.00

Continued on next page

Gene Ontology Identification Number		Raw pValue		Corrected pValue*		% Associated Genes	Nr. Genes
		Term	Group	Term	Group		
GO:0034446	substrate adhesion-dependent cell spreading	2.02E-05	3.89E-09	3.44E-04	7.40E-08	5.45	6.00
GO:0043149	stress fiber assembly	3.54E-06	3.89E-09	1.03E-04	7.40E-08	5.56	7.00
GO:0110020	regulation of actomyosin structure organization	2.85E-06	3.89E-09	9.99E-05	7.40E-08	5.74	7.00
GO:0001954	positive regulation of cell-matrix adhesion	3.92E-05	3.89E-09	3.53E-04	7.40E-08	6.67	5.00
GO:0002040	sprouting angiogenesis	4.14E-06	5.75E-09	1.12E-04	1.04E-07	5.43	7.00
GO:0043534	blood vessel endothelial cell migration	6.86E-08	5.75E-09	4.26E-06	1.04E-07	6.00	9.00
GO:1903670	regulation of sprouting angiogenesis	5.68E-05	5.75E-09	2.84E-04	1.04E-07	6.17	5.00
GO:0002042	cell migration involved in sprouting angiogenesis	3.01E-05	5.75E-09	3.91E-04	1.04E-07	7.04	5.00
GO:0016101	diterpenoid metabolic process	2.85E-06	6.38E-09	9.99E-05	1.08E-07	5.74	7.00
GO:0006695	cholesterol biosynthetic process	7.24E-06	6.38E-09	1.74E-04	1.08E-07	6.52	6.00
GO:0015914	phospholipid transport	3.45E-06	6.38E-09	1.07E-04	1.08E-07	7.41	6.00
GO:0006641	triglyceride metabolic process	1.02E-09	6.38E-09	8.26E-08	1.08E-07	7.75	10.00
GO:0097006	regulation of plasma lipoprotein particle levels	5.88E-10	6.38E-09	5.00E-08	1.08E-07	8.20	10.00
GO:0046503	glycerolipid catabolic process	9.33E-08	6.38E-09	5.41E-06	1.08E-07	9.46	7.00
GO:0042632	cholesterol homeostasis	1.11E-09	6.38E-09	8.87E-08	1.08E-07	9.57	9.00
GO:0071825	protein-lipid complex subunit organization	3.25E-12	6.38E-09	2.95E-10	1.08E-07	13.70	10.00
GO:0019433	triglyceride catabolic process	7.56E-08	6.38E-09	4.46E-06	1.08E-07	13.95	6.00

Continued on next page

Gene Ontology Identification Number		Raw pValue		Corrected pValue*		% Associated Genes	Nr. Genes
		Term	Group	Term	Group		
GO:0034369	plasma lipoprotein particle remodeling	6.22E-10	6.38E-09	5.22E-08	1.08E-07	18.92	7.00
GO:0034378	chylomicron assembly	2.44E-08	6.38E-09	1.71E-06	1.08E-07	27.78	5.00
GO:0007219	Notch signaling pathway	4.10E-08	4.10E-08	2.74E-06	6.55E-07	4.53	11.00
GO:0008593	regulation of Notch signaling pathway	4.56E-05	4.10E-08	3.19E-04	6.55E-07	4.72	6.00
GO:0031099	regeneration	4.84E-08	4.84E-08	3.15E-06	7.26E-07	4.45	11.00
GO:0010001	glial cell differentiation	6.97E-08	6.97E-08	4.25E-06	9.76E-07	4.30	11.00
GO:0048708	astrocyte differentiation	1.17E-05	6.97E-08	2.58E-04	9.76E-07	6.00	6.00
GO:0051057	positive regulation of small GTPase mediated signal transduction	4.22E-07	4.22E-07	2.07E-05	5.49E-06	7.61	7.00
GO:1902003	regulation of amyloid-beta formation	5.43E-07	7.45E-07	2.55E-05	8.93E-06	15.63	5.00
GO:1902992	negative regulation of amyloid precursor protein catabolic process	7.39E-08	7.45E-07	4.44E-06	8.93E-06	22.73	5.00
GO:0002920	regulation of humoral immune response	1.09E-06	1.09E-06	4.46E-05	1.20E-05	5.23	8.00
GO:0090288	negative regulation of cellular response to growth factor stimulus	3.03E-05	2.62E-06	3.63E-04	2.09E-05	4.00	7.00
GO:0090101	negative regulation of transmembrane receptor protein serine/threonine kinase signaling pathway	7.11E-06	2.62E-06	1.78E-04	2.09E-05	5.00	7.00
GO:0030512	negative regulation of transforming growth factor beta receptor signaling pathway	6.39E-05	2.62E-06	2.56E-04	2.09E-05	6.02	5.00

Continued on next page

Gene Ontology Identification Number		Raw pValue		Corrected pValue*		% Associated Genes	Nr. Genes
		Term	Group	Term	Group		
GO:0019985	translesion synthesis	3.06E-07	2.62E-06	1.59E-05	2.09E-05	11.11	6.00
GO:0006296	nucleotide-excision repair, DNA incision, 5'-to lesion	3.13E-06	2.62E-06	1.00E-04	2.09E-05	11.11	5.00
GO:0061418	regulation of transcription from RNA polymerase II promoter in response to hypoxia	1.49E-07	2.62E-06	8.20E-06	2.09E-05	12.50	6.00
GO:0008361	regulation of cell size	7.27E-07	2.11E-06	3.27E-05	2.11E-05	4.55	9.00
GO:0001952	regulation of cell-matrix adhesion	1.84E-06	2.11E-06	6.97E-05	2.11E-05	4.88	8.00
GO:0061387	regulation of extent of cell growth	3.47E-05	2.11E-06	3.47E-04	2.11E-05	4.96	6.00
GO:0048041	focal adhesion assembly	2.36E-05	2.11E-06	3.30E-04	2.11E-05	5.31	6.00
GO:0048844	artery morphogenesis	2.37E-06	2.37E-06	8.53E-05	2.13E-05	7.89	6.00
GO:0045824	negative regulation of innate immune response	1.32E-05	1.32E-05	2.50E-04	7.90E-05	8.33	5.00
GO:0046887	positive regulation of hormone secretion	1.22E-05	1.22E-05	2.56E-04	8.53E-05	4.61	7.00
GO:0051341	regulation of oxidoreductase activity	3.31E-05	3.31E-05	3.64E-04	1.66E-04	5.00	6.00
GO:0050999	regulation of nitric-oxide synthase activity	1.95E-05	3.31E-05	3.52E-04	1.66E-04	7.69	5.00
GO:0042246	tissue regeneration	4.74E-05	4.74E-05	2.84E-04	1.90E-04	6.41	5.00
GO:0003279	cardiac septum development	6.68E-05	6.68E-05	2.00E-04	2.00E-04	4.41	6.00

Continued on next page

Gene Ontology Identification Number		Raw pValue		Corrected pValue*		% Associated Genes	Nr. Genes
		Term	Group	Term	Group		
GO:0031100	animal organ regeneration	3.76E-04	3.76E-04	3.76E-04	3.76E-04	4.13	5.00
GO:0042310	vasoconstriction	2.22E-04	2.22E-04	4.44E-04	4.44E-04	4.63	5.00

Note: The figure shows the enriched Cluster 1, derived from the initial selection of genes. Cluster 1 depicts the interacting genes and the type of interaction as shown in the figure legend. The Bar Chart associated with cluster 1 reports the percentage of genes belonging to a specific biological process (from Gene Ontology database) included in the pathway under analysis. It also shows the associations levels of biological processes with the cluster under analysis. The pie chart shows the overall degree of association of main biological processes involved with the cluster under investigation. To note, both the bar and pie chart report the top 25 biological processes. The table summarizes the details regarding the pathway analysis and shows all of the associated biological processes linked with the cluster under investigation.

Cluster 2. Informative Data.

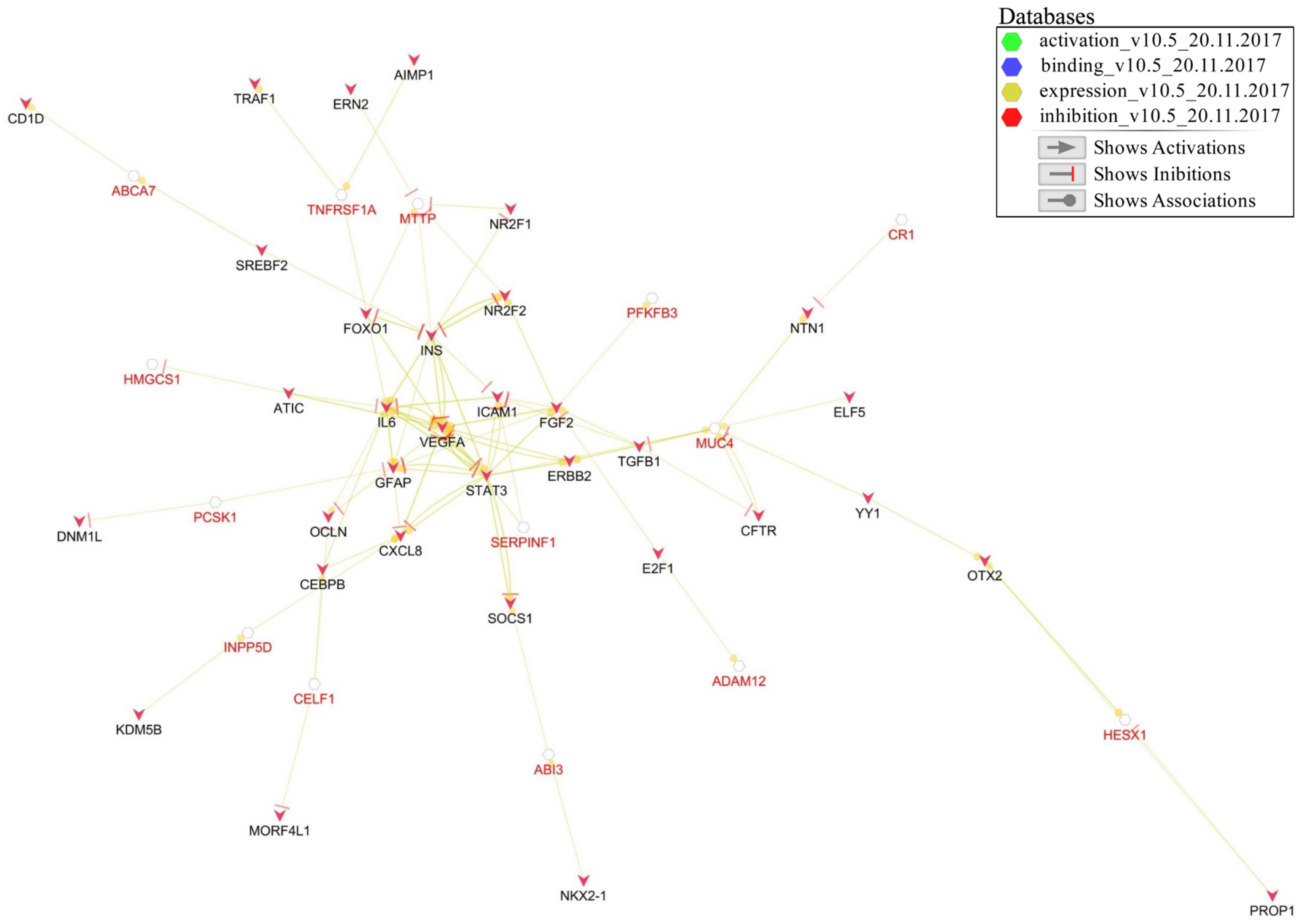

Cluster 2. Informative Data.

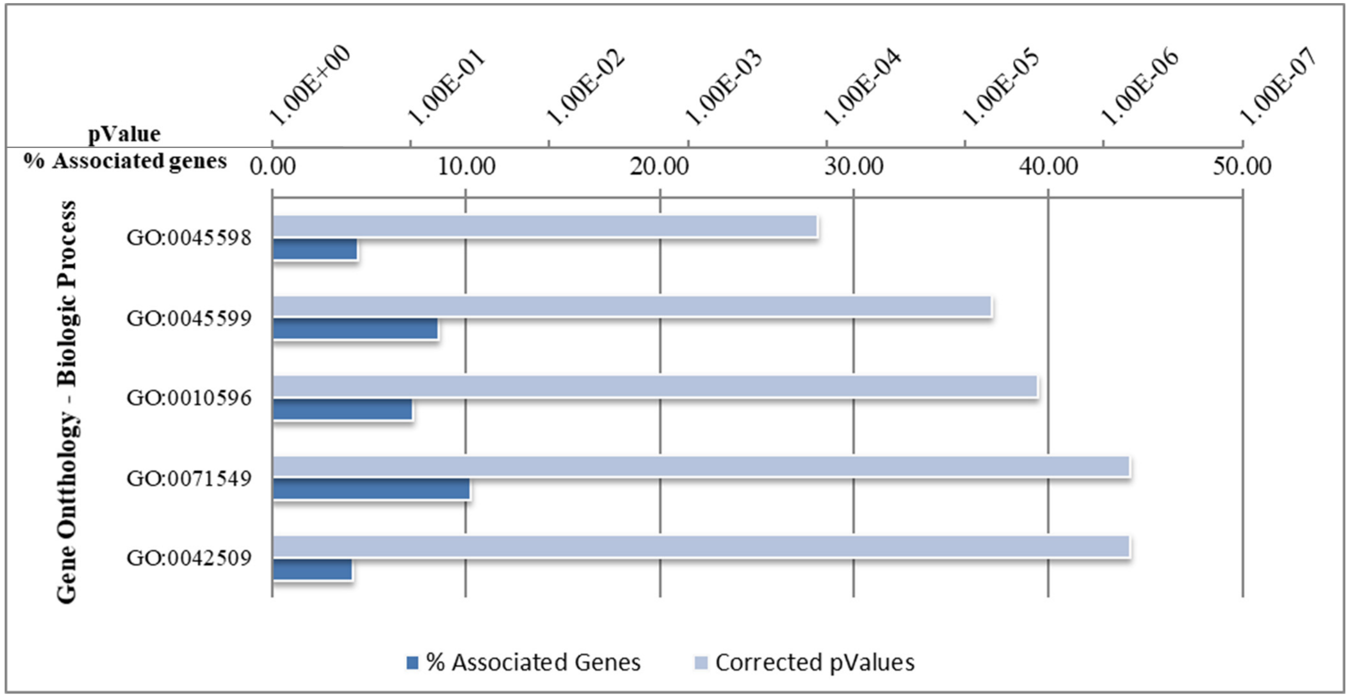

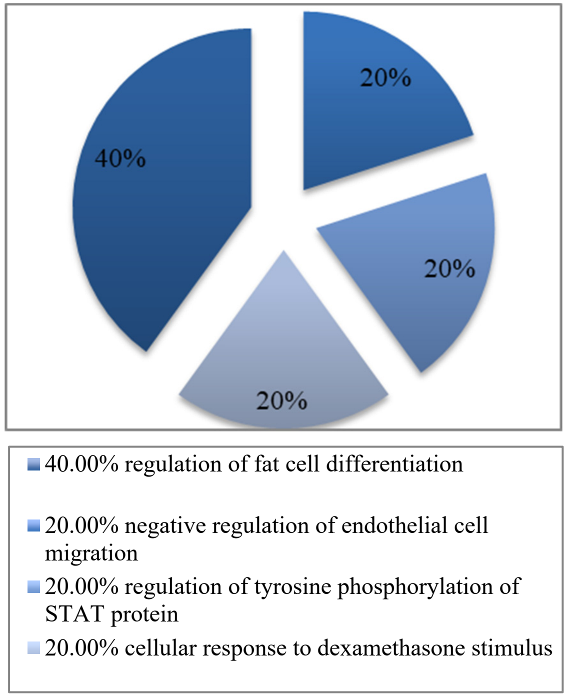

Cluster 2. Informative Data.

Gene Ontology Identification Number		Raw pValue		Corrected pValue*		% Associated Genes	Nr. Genes
		Term	Group	Term	Group		
GO:0045598	regulation of fat cell differentiation	1.61E-07	8.06E-07	1.61E-07	6.45E-07	4.46	7.00
GO:0045599	negative regulation of fat cell differentiation	4.09E-07	1.64E-06	1.61E-07	6.45E-07	8.62	5.00
GO:0010596	negative regulation of endothelial cell migration	9.83E-07	2.95E-06	9.83E-07	2.95E-06	7.25	5.00
GO:0071549	cellular response to dexamethasone stimulus	3.18E-06	6.36E-06	3.18E-06	6.36E-06	10.26	4.00
GO:0042509	regulation of tyrosine phosphorylation of STAT protein	1.15E-04	1.15E-04	1.15E-04	1.15E-04	4.17	4.00

The figure shows the enriched Cluster 2, derived from the initial selection of genes, after exclusion of the genes involved in cluster 1. Cluster 2 depicts the interacting genes and the type of interaction as shown in the figure legend. The Bar Chart associated with cluster 2 reports the percentage of genes belonging to a specific biological process (from Gene Ontology database) included in the pathway under analysis. It also shows the associations levels of biological processes with the cluster under analysis. The pie chart shows the overall degree of association of main biological processes involved with the cluster under investigation. Cluster 2 genes seems to be mainly implicated with fat regulative processes. To note GO:0045599 - negative regulation of fat cell differentiation is a sub process of GO:0045598 - regulation of fat cell differentiation. Only main biological processes are represented in the pie chart. The table summarizes the details regarding the pathway analysis and shows all of the associated biological processes linked with the cluster under investigation.

Cluster 3. Informative Data.

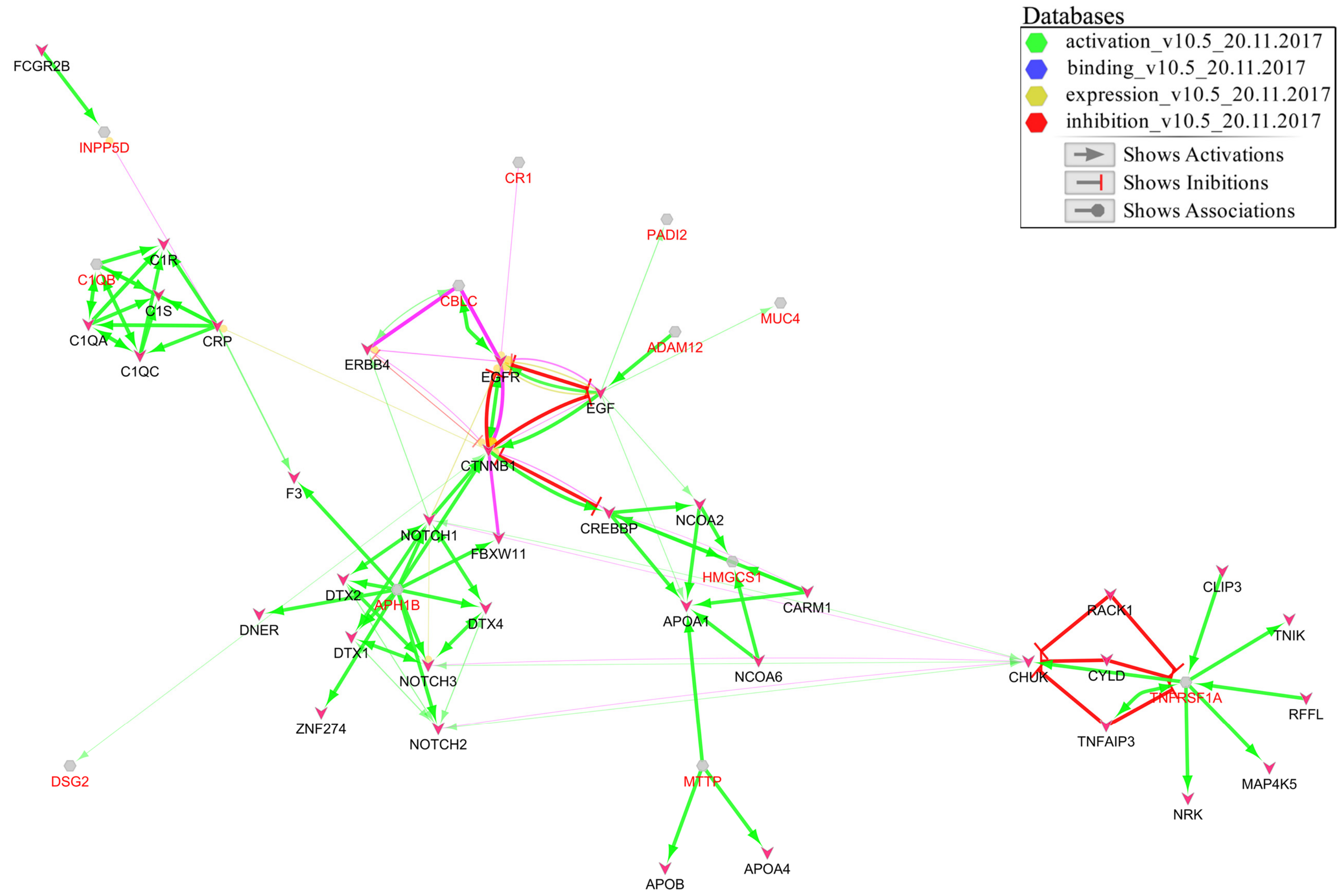

Cluster 3. Informative Data.

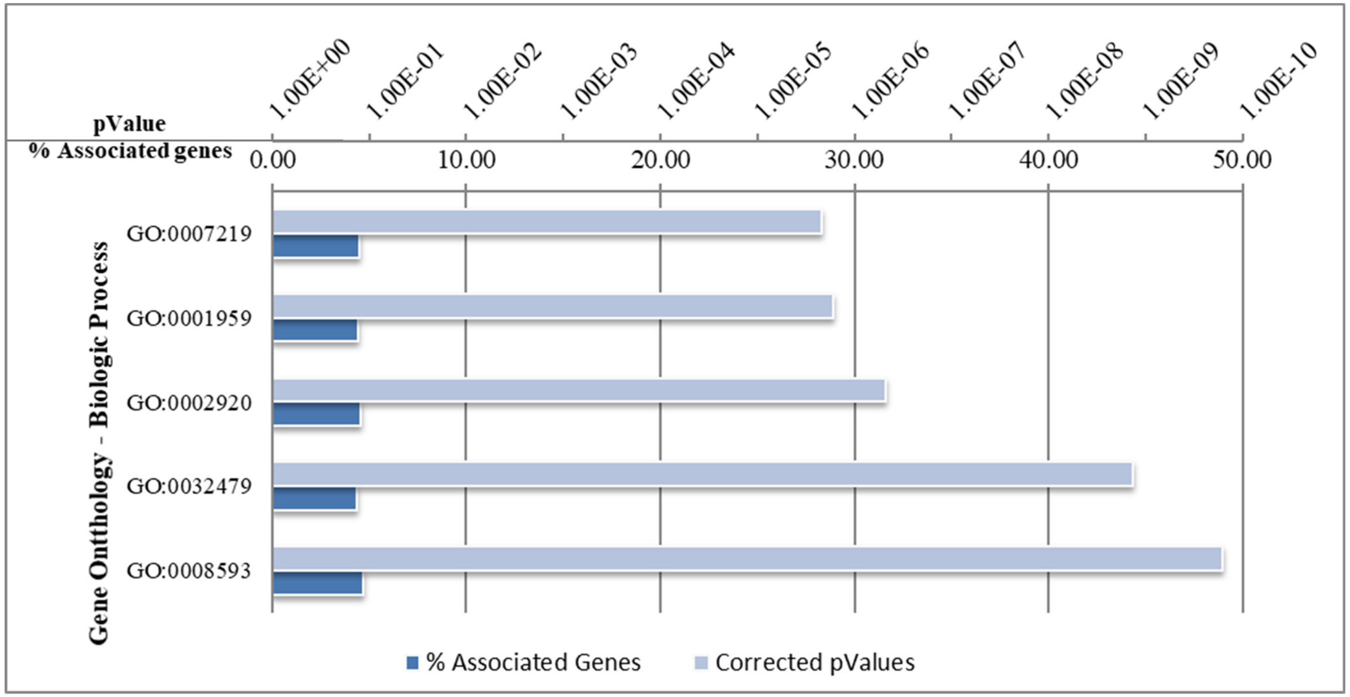

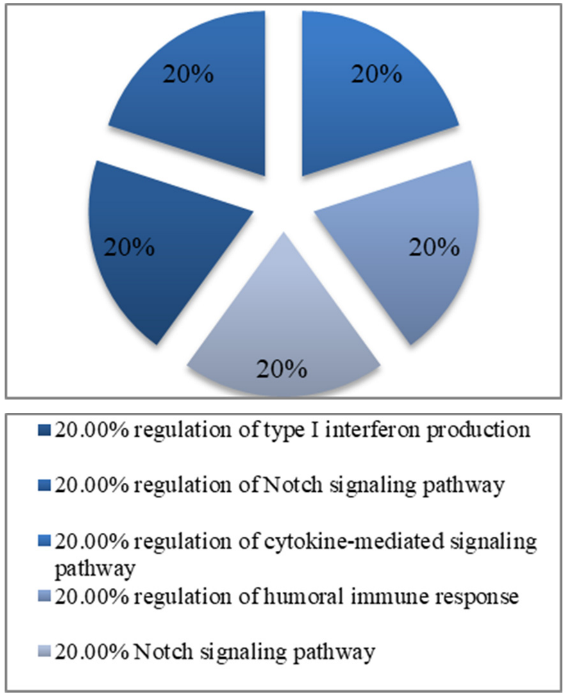

Cluster 3. Informative Data.

Gene Ontology Identification Number		Raw pValue		Corrected pValue*		% Associated Genes	Nr. Genes
		Term	Group	Term	Group		
GO:0007219	Notch signaling pathway	3.26E-11	3.26E-11	1.63E-10	1.63E-10	4.53	11
GO:0001959	regulation of cytokine-mediated signaling pathway	3.40E-10	3.40E-10	1.36E-09	1.36E-09	4.44	10
GO:0002920	regulation of humoral immune response	1.57E-07	1.57E-07	4.71E-07	4.71E-07	4.58	7
GO:0032479	regulation of type I interferon production	1.68E-06	1.68E-06	1.68E-06	1.68E-06	4.38	6
GO:0008593	regulation of Notch signaling pathway	1.08E-06	1.08E-06	2.15E-06	2.15E-06	4.72	6

The figure shows the enriched Cluster 3, derived from the initial selection of genes, after exclusion of previous clusters. Cluster 3 depicts the interacting genes and the type of interaction as shown in the figure legend. The Bar Chart associated with cluster 3 reports the percentage of genes belonging to a specific biological process (from Gene Ontology database) included in the pathway under analysis. It also shows the associations levels of biological processes with the cluster under analysis. The pie chart shows the overall degree of association of main biological processes involved with the cluster under investigation. The table summarizes the details regarding the pathway analysis and shows all of the associated biological processes linked with the cluster under investigation.

Cluster 4. Informative Data.

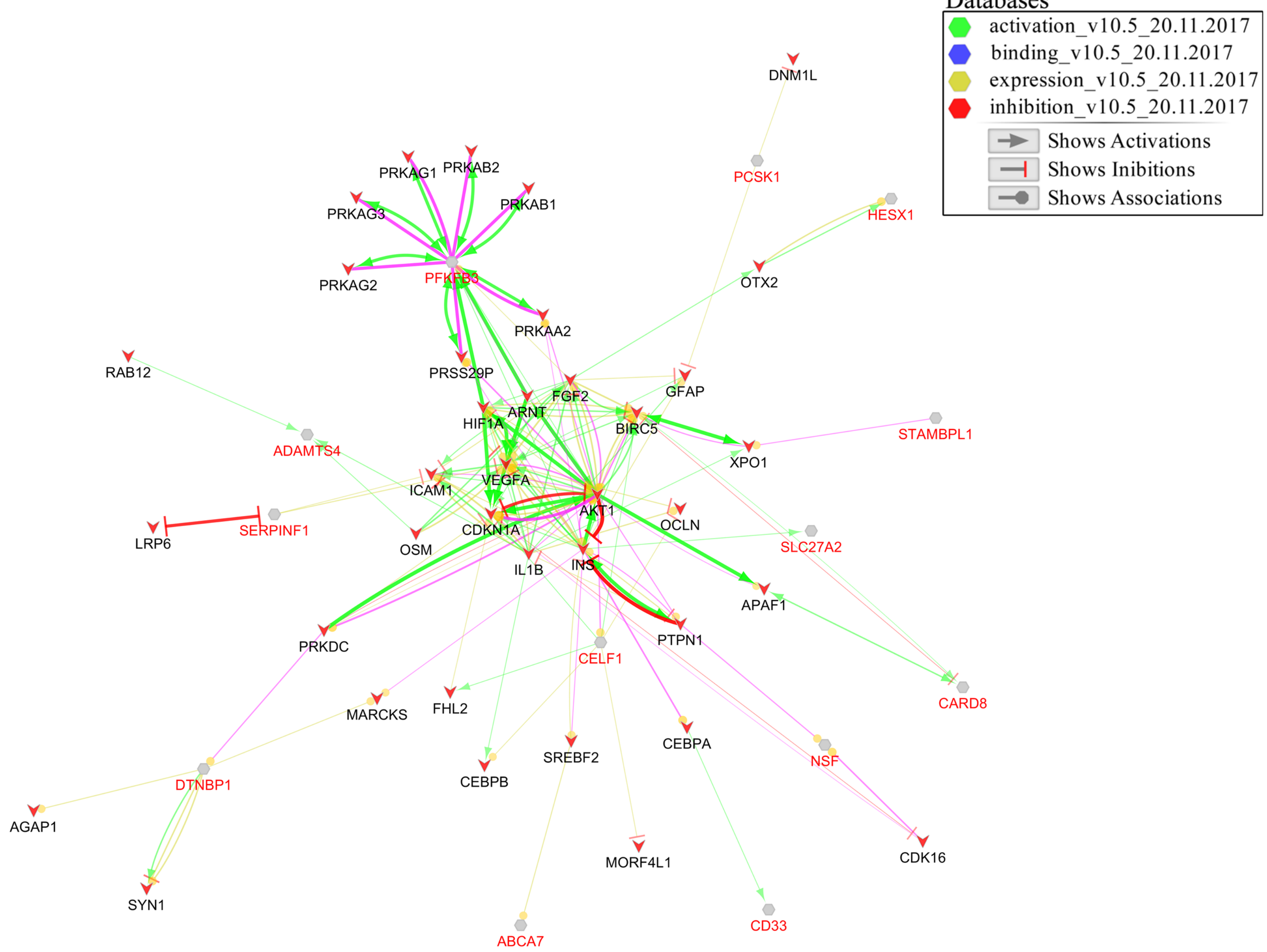

Cluster 4. Informative Data.

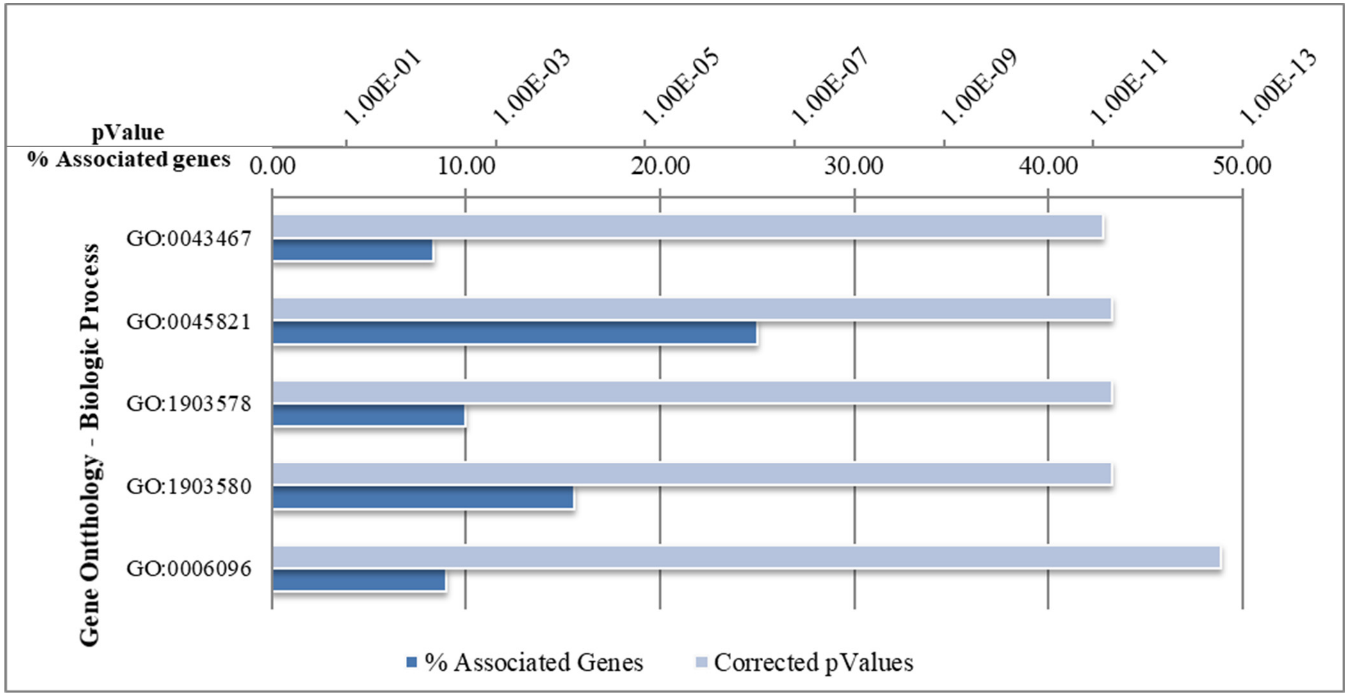

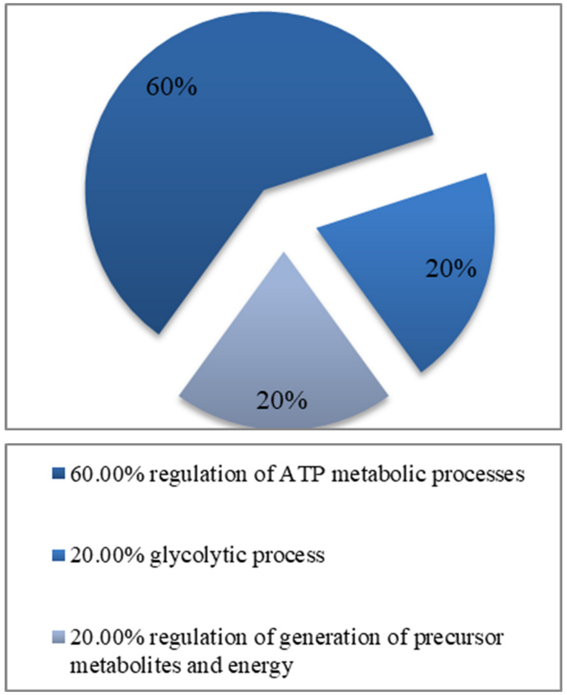

Cluster 4. Informative Data.

Gene Ontology Identification Number		Raw pValue		Corrected pValue*		% Associated Genes	Nr. Genes
		Term	Group	Term	Group		
GO:0043467	regulation of generation of precursor metabolites and energy	6.56E-14	6.56E-14	3.28E-13	1.97E-13	8.33	11
GO:0045821	positive regulation of glycolytic process	4.45E-11	2.83E-12	4.45E-11	5.66E-12	25.00	6
GO:1903578	regulation of ATP metabolic process	2.83E-12	2.83E-12	1.13E-11	5.66E-12	10.00	9
GO:1903580	positive regulation of ATP metabolic process	3.53E-11	2.83E-12	7.06E-11	5.66E-12	15.56	7
GO:0006096	glycolytic process	7.46E-12	7.46E-12	2.24E-11	7.46E-12	9.00	9

The figure shows the enriched Cluster 4, derived from the initial selection of genes, after exclusion of previous clusters. Cluster 4 depicts the interacting genes and the type of interaction as shown in the figure legend. The Bar Chart associated with cluster 4 reports the percentage of genes belonging to a specific biological process (from Gene Ontology database) included in the pathway under analysis. It also shows the associations levels of biological processes with the cluster under analysis. The pie chart shows the overall degree of association of main biological processes involved with the cluster under investigation. To note, only main biological processes are represented in the pie chart. The table summarizes the details regarding the pathway analysis and shows all of the associated biological processes linked with the cluster under investigation.

Cluster 5. Informative Data.

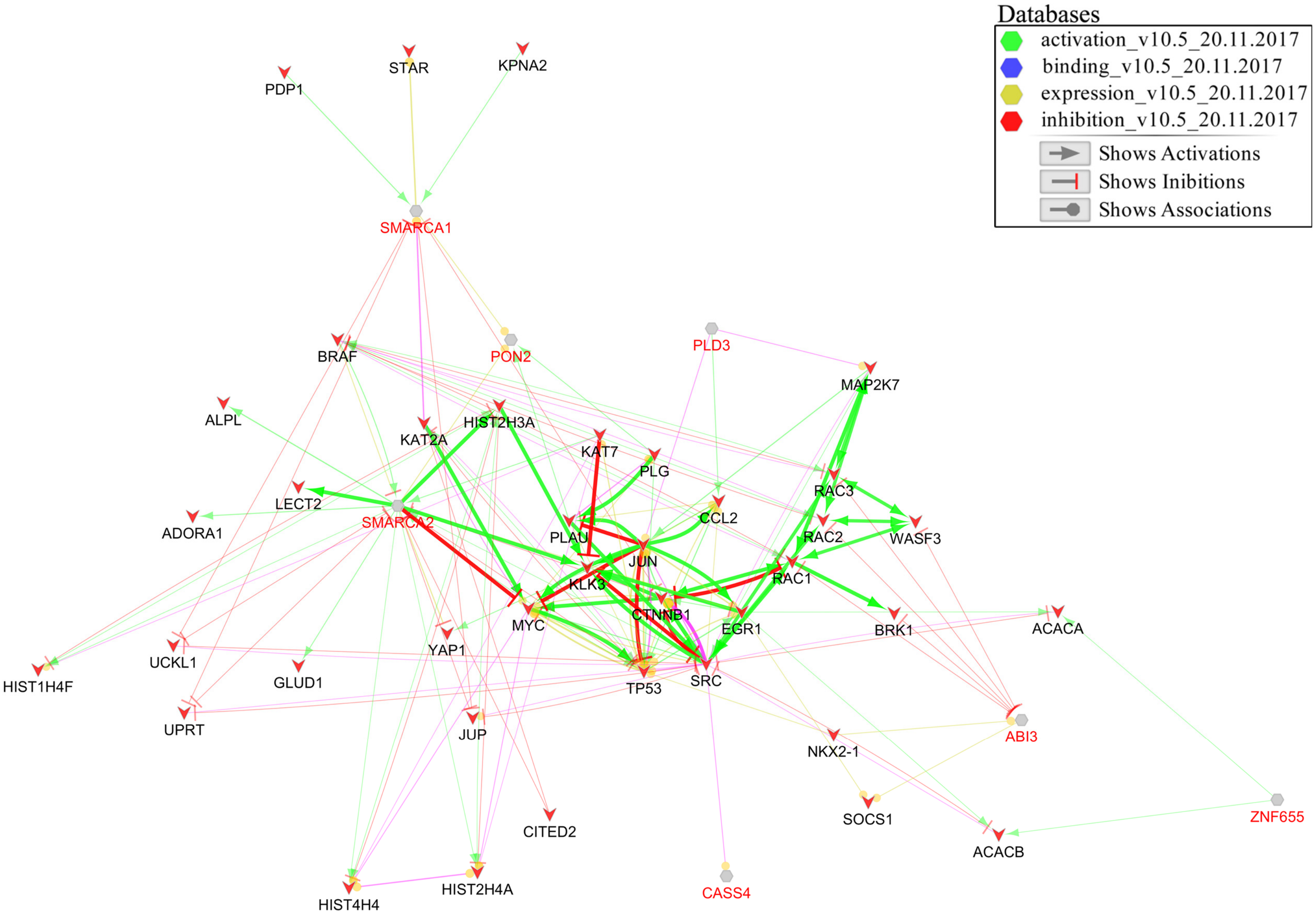

Cluster 5. Informative Data.

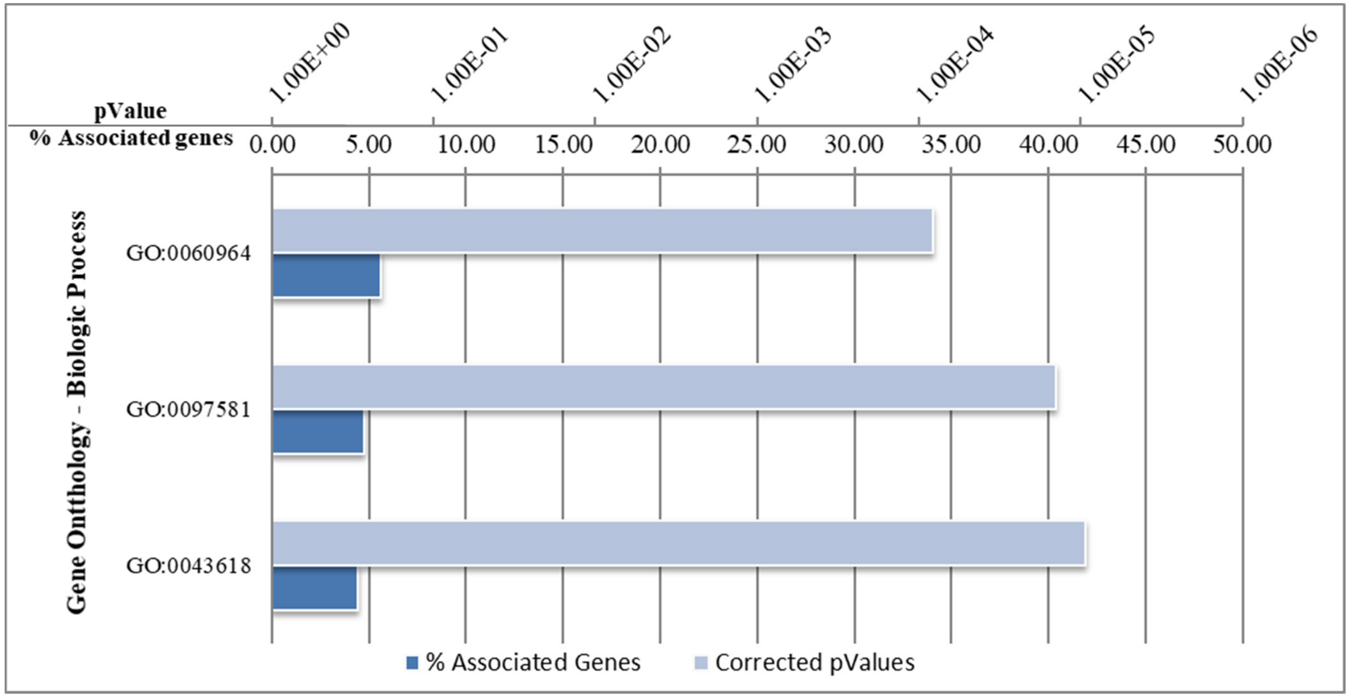

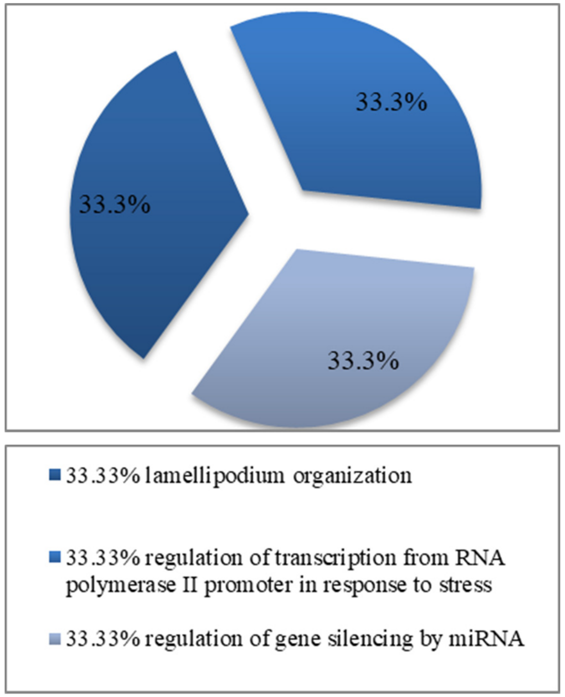

Cluster 5. Informative Data.

	Gene Ontology Identification Number	Raw pValue		Corrected pValue*		% Associated Genes	Nr. Genes
		Term	Group	Term	Group		
GO:0060964	regulation of gene silencing by miRNA	3.13E-06	3.13E-06	9.40E-06	9.40E-06	5.62	5
GO:0097581	lamellipodium organization	7.07E-06	7.07E-06	1.41E-05	1.41E-05	4.76	5
GO:0043618	regulation of transcription from RNA polymerase II promoter in response to stress	8.23E-05	8.23E-05	8.23E-05	8.23E-05	4.44	4

The figure shows the enriched Cluster 5, derived from the initial selection of genes, after exclusion of previous clusters. Cluster 5 depicts the interacting genes and the type of interaction as shown in the figure legend. The Bar Chart associated with cluster 5 reports the percentage of genes belonging to a specific biological process (from Gene Ontology database) included in the pathway under analysis. It also shows the associations levels of biological processes with the cluster under analysis. The pie chart shows the overall degree of association of main biological processes involved with the cluster under investigation. The table summarizes the details regarding the pathway analysis and shows all of the associated biological processes linked with the cluster under investigation.

Cluster 6. Informative Data.

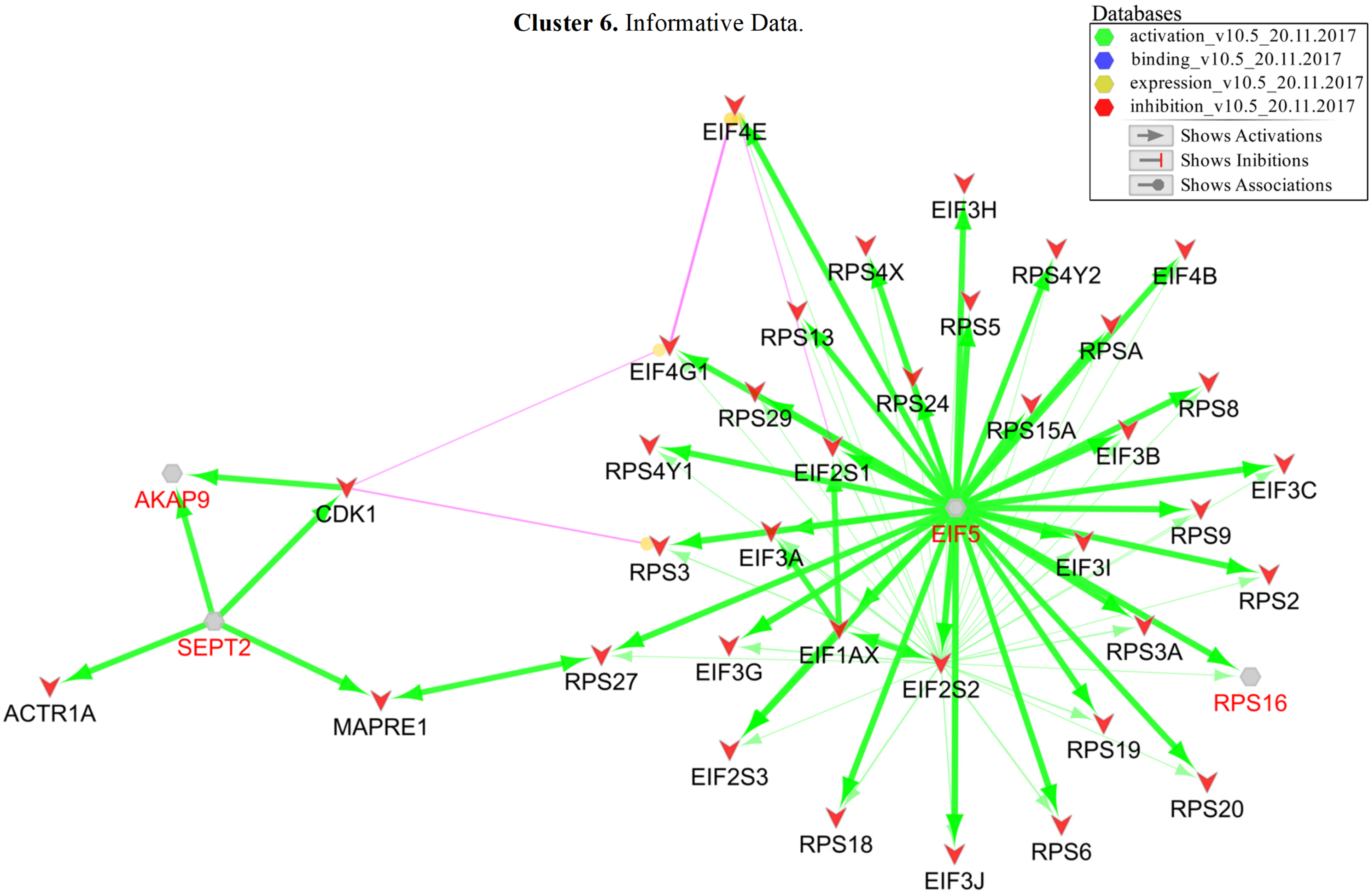

Cluster 6. Informative Data.

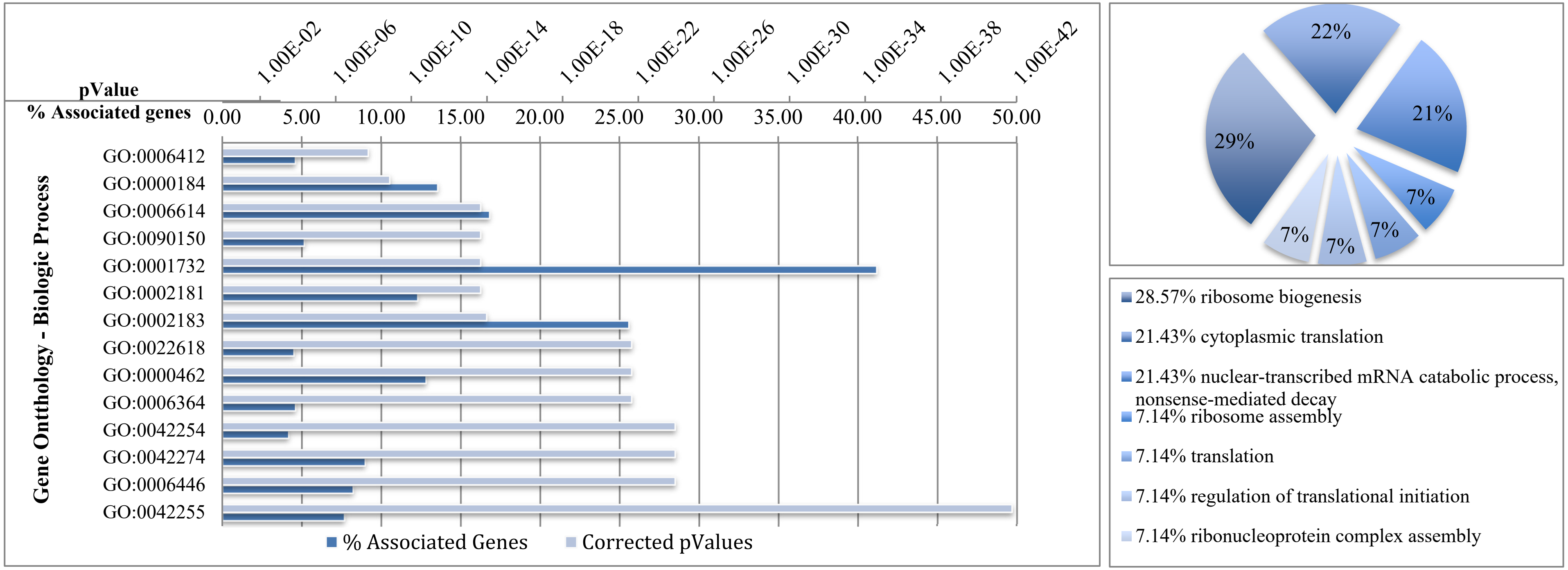

Cluster 6. Informative Data.

Gene Ontology Identification Number		Raw pValue		Corrected pValue*		% Associated Genes	Nr. Genes
		Term	Group	Term	Group		
GO:0006412	translation	2.43E-43	2.43E-43	3.40E-42	1.70E-42	4.59	35
GO:0000184	nuclear-transcribed mRNA catabolic process, nonsense-mediated decay	1.30E-33	1.85E-25	1.69E-32	1.11E-24	13.55	21
GO:0006614	SRP-dependent cotranslational protein targeting to membrane	3.68E-32	1.85E-25	4.42E-31	1.11E-24	16.81	19
GO:0090150	establishment of protein localization to membrane	2.86E-23	1.85E-25	3.15E-22	1.11E-24	5.15	20
GO:0001732	formation of cytoplasmic translation initiation complex	3.01E-15	4.41E-23	2.11E-14	2.21E-22	41.18	7
GO:0002181	cytoplasmic translation	4.41E-23	4.41E-23	4.41E-22	2.21E-22	12.30	15
GO:0002183	cytoplasmic translational initiation	8.20E-21	4.41E-23	7.38E-20	2.21E-22	25.58	11
GO:0022618	ribonucleoprotein complex assembly	2.56E-15	2.56E-15	2.05E-14	1.02E-14	4.50	14
GO:0000462	maturation of SSU-rRNA from tricistronic rRNA transcript	2.32E-08	7.16E-15	2.32E-08	2.15E-14	12.82	5
GO:0006364	rRNA processing	3.07E-12	7.16E-15	1.53E-11	2.15E-14	4.60	11
GO:0042254	ribosome biogenesis	7.16E-15	7.16E-15	4.30E-14	2.15E-14	4.18	14
GO:0042274	ribosomal small subunit biogenesis	1.80E-11	7.16E-15	7.19E-11	2.15E-14	8.99	8

Continued on next page

Gene Ontology Identification Number		Raw pValue		Corrected pValue*		% Associated Genes	Nr. Genes
		Term	Group	Term	Group		
GO:0006446	regulation of translational initiation	6.85E-10	6.85E-10	2.05E-09	1.37E-09	8.24	7
GO:0042255	ribosome assembly	1.90E-08	1.90E-08	3.79E-08	1.90E-08	7.69	6

The figure shows the enriched Cluster 6, derived from the initial selection of genes, after exclusion of previous clusters. Cluster 6 depicts the interacting genes and the type of interaction as shown in the figure legend. The Bar Chart associated with cluster 6 reports the percentage of genes belonging to a specific biological process (from Gene Ontology database) included in the pathway under analysis. It also shows the associations levels of biological processes with the cluster under analysis. The pie chart shows the overall degree of association of main biological processes involved with the cluster under investigation. To note, only main biological processes are represented in the pie chart. The table summarizes the details regarding the pathway analysis and shows all of the associated biological processes linked with the cluster under investigation.

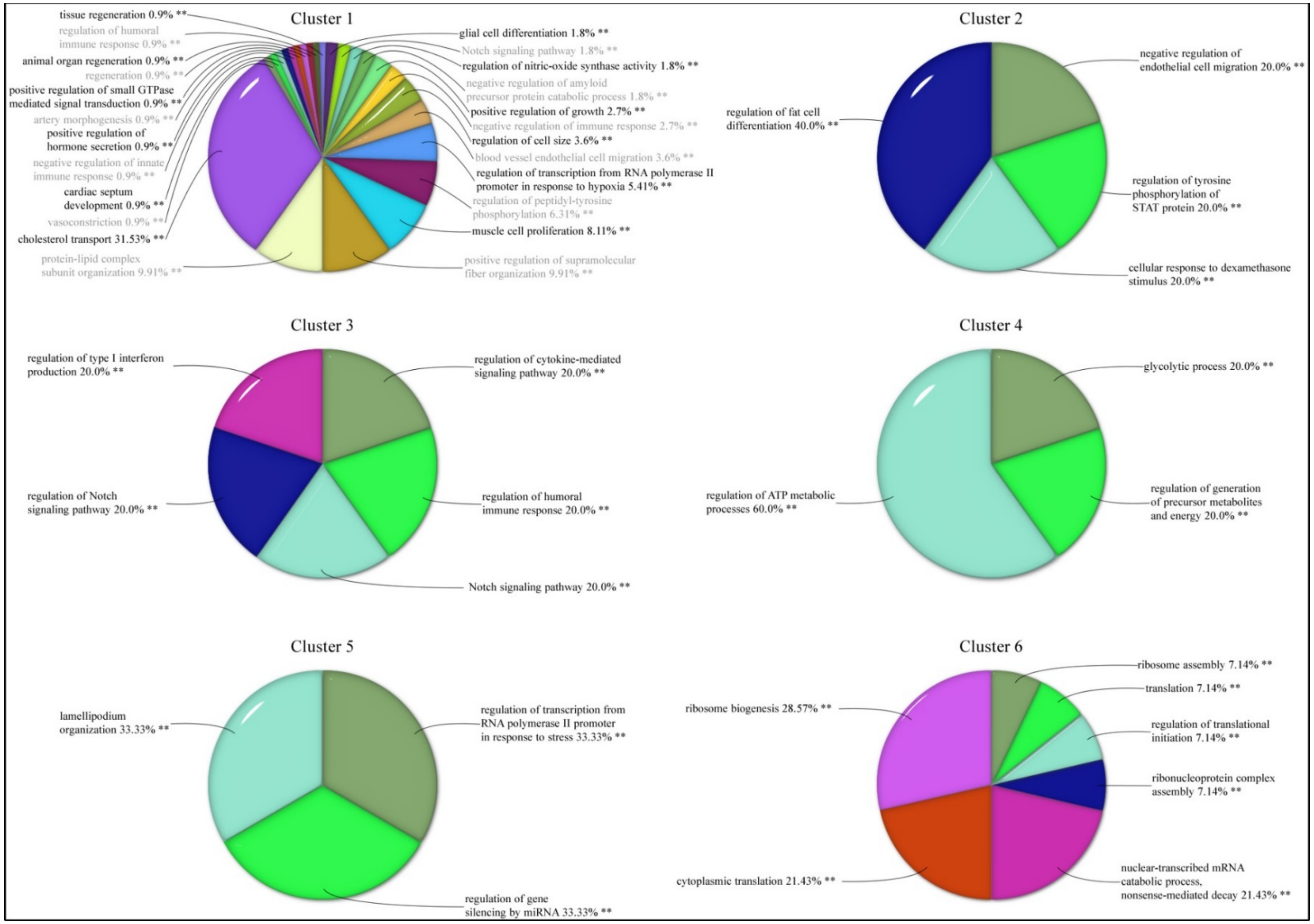

Supplementary Figure 1. Reports the overall summary of the biological processes associated (p <0.05 with Bonferroni step down correction) with the clusters enriched from the initial list of genes (see Table1). Enrichment was based on STRING database (STRING-ACTIONS_v10.5) while pathway recognition used Gene Ontology Database (GO_BiologicalProcess-EBI-UniProt-GOA). Both analyses were performed on Cytoscape software [22], through ClueGO and CluePedia plugins [23].

References

1. Kumar A, Tsao JW (2018) *Alzheimer Disease*, in *StatPearls*, Treasure Island (FL).
2. Grossberg GT, Desai AK (2003) Management of Alzheimer's disease. *J Gerontol A Biol Sci Med Sci* 58: 331–353.
3. Watkins PB, Zimmerman HJ, Knapp MJ, et al. (1994) Hepatotoxic effects of tacrine administration in patients with Alzheimer's disease. *JAMA* 271: 992–998.
4. Winblad B, Wimo A, Engedal K, et al. (2006) 3-year study of donepezil therapy in Alzheimer's disease: effects of early and continuous therapy. *Dement Geriatr Cogn Disord* 21: 353–363.
5. Carrier L (1999) Donepezil and paroxetine: possible drug interaction. *J Am Geriatr Soc* 47: 1037.
6. Cacabelos R (2007) Donepezil in Alzheimer's disease: from conventional trials to pharmacogenetics. *Neuropsychiatr Dis Treat* 3: 303–333.
7. Grossberg GT, Stahelin HB, Messina JC, et al. (2000) Lack of adverse pharmacodynamic drug interactions with rivastigmine and twenty-two classes of medications. *Int J Geriatr Psychiatry* 15: 242–247.
8. Farlow MR, Hake A, Messina J, et al. (2001) Response of patients with Alzheimer disease to rivastigmine treatment is predicted by the rate of disease progression. *Arch Neurol* 58: 417–422.
9. Raskind MA, Peskind ER, Wessel T, et al. (2000) Galantamine in AD: a 6-month randomized, placebo-controlled trial with a 6-month extension. The Galantamine USA-1 Study Group. *Neurology* 54: 2261–2268.
10. Winblad B, Poritis N (1999) Memantine in severe dementia: results of the 9M-Best Study (Benefit and efficacy in severely demented patients during treatment with memantine). *Int J Geriatr Psychiatry* 14: 135–146.
11. Verrico MM, Nace DA, Towers AL (2000) Fulminant chemical hepatitis possibly associated with donepezil and sertraline therapy. *J Am Geriatr Soc* 48: 1659–1663.
12. Kruger J, Moilanen V, Majamaa K, et al. (2012) Molecular genetic analysis of the APP, PSEN1, and PSEN2 genes in Finnish patients with early-onset Alzheimer disease and frontotemporal lobar degeneration. *Alzheimer Dis Assoc Disord* 26: 272–276.
13. Montine KS, Olson SJ, Amarnath V, et al. (1997) Immunohistochemical detection of 4-hydroxy-2-nonenal adducts in Alzheimer's disease is associated with inheritance of APOE4. *Am J Pathol* 150: 437–443.
14. Farrer LA, Cupples LA, Haines JL, et al. (1997) Effects of age, sex, and ethnicity on the association between apolipoprotein E genotype and Alzheimer disease. A meta-analysis. APOE and Alzheimer Disease Meta Analysis Consortium. *JAMA* 278: 1349–1356.
15. Bis JC, Jian X, Kunkle BW, et al. (2020) Whole exome sequencing study identifies novel rare and common Alzheimer's-Associated variants involved in immune response and transcriptional regulation. *Mol Psychiatry* 25: 1859–1875.
16. Yao F, Zhang K, Zhang Y, et al. (2018) Identification of blood biomarkers for Alzheimer's disease through computational prediction and experimental validation. *Front Neurol* 9: 1158.
17. Han ZJ, Xue WW, Tao L, et al. (2018) Identification of novel immune-relevant drug target genes for Alzheimer's disease by combining ontology inference with network analysis. *CNS Neurosci Ther* 24: 1253–1263.

18. Jansen IE, Savage JE, Watanabe K, et al. (2019) Genome-wide meta-analysis identifies new loci and functional pathways influencing Alzheimer's disease risk. *Nat Genet* 51: 404–413.
19. Beecham GW, Vardarajan B, Blue E, et al. (2018) Rare genetic variation implicated in non-Hispanic white families with Alzheimer disease. *Neurol Genet* 4: e286.
20. Sancesario GM, Bernardini S (2018) Alzheimer's disease in the omics era. *Clin Biochem* 59: 9–16.
21. Rustenhoven J, Smith AM, Smyth LC, et al. (2018) PU.1 regulates Alzheimer's disease-associated genes in primary human microglia. *Mol Neurodegener* 13: 44.
22. Shannon P, Markiel A, Ozier O, et al. (2003) Cytoscape: a software environment for integrated models of biomolecular interaction networks. *Genome Res* 13: 2498–2504.
23. Bindea G, Mlecnik B, Hackl H, et al. (2009) ClueGO: a Cytoscape plug-in to decipher functionally grouped gene ontology and pathway annotation networks. *Bioinformatics* 25: 1091–1093.

AIMS Press

© 2021 the Author(s), licensee AIMS Press. This is an open access article distributed under the terms of the Creative Commons Attribution License (http://creativecommons.org/licenses/by/4.0)
